# Contamination of the marine environment by Antarctic research stations: Monitoring marine pollution at Casey station from 1997 to 2015

**DOI:** 10.1371/journal.pone.0288485

**Published:** 2023-08-09

**Authors:** Jonathan S. Stark, Glenn J. Johnstone, Catherine King, Tania Raymond, Allison Rutter, Scott C. Stark, Ashley T. Townsend

**Affiliations:** 1 Australian Antarctic Division, East Antarctic Monitoring Program, Kingston, Tasmania, Australia; 2 Analytical Services Unit, Queens University, Kingston, Ontario, Canada; 3 Central Science Laboratory, College of Sciences and Engineering, University of Tasmania, Hobart, Tasmania, Australia; The University of Auckland - City Campus: University of Auckland, NEW ZEALAND

## Abstract

The contamination of the marine environment surrounding coastal Antarctic research stations remains insufficiently understood in terms of its extent, persistence, and characteristics. We investigated the presence of contaminants in marine sediments near Casey Station, located in the Windmill Islands of East Antarctica, during the period spanning from 1997 to 2015. Metals, hydrocarbons, PBDEs, PCBs, and nutrients were measured in sediments at anthropogenically disturbed sites, including the wastewater outfall, the wharf area, two former waste disposal sites, and various control locations. Sampling was carried out at three spatial scales: Locations, which were generally kilometres apart and formed the primary scale for comparison; Sites, which were 100 meters apart within each location; and Plots, which were 10 meters apart within each site. Consistently higher concentrations of most contaminants, and in some cases nutrients, were observed at disturbed locations. Some locations also exhibited an increase in contaminant concentrations over time. The spatial distribution of sediment properties (such as grain size and organic matter) and contaminants displayed intricate patterns of variation. Variation in grain size depended on the size category, with fine grains (e.g., <63 μm) showing the greatest variation at the Location scale, while coarse grains exhibited minimal variation at this scale. Contaminant levels demonstrated significant differences between Locations, accounting for approximately 55% of the overall variation for metals, while the variation within the 10-meter scale generally exceeded that within the 100-meter scale. Residual variation among replicate samples was also very high, demonstrating the need for adequate replication in studies of sediments and contaminants around stations. Some contaminants exceeded international guidelines for sediment quality, including metals, hydrocarbons, and PCBs. We conclude that Antarctic research stations such as Casey are likely to pose a moderate level of long-term ecological risk to local marine ecosystems through marine pollution. However, contamination is expected to be confined to areas in close proximity to the stations, although its extent and concentration are anticipated to increase with time. Raising awareness of the contamination risks associated with Antarctic stations and implementing monitoring programs for marine environments adjacent to these stations can contribute to informed decision-making and the improvement of environmental management practices in Antarctica.

## Introduction

The human ‘footprint’ and spatial extent of human activities and associated impacts in Antarctica, continues to grow as national Antarctic programs establish, expand, modernise and rebuild stations. There are currently 112 scientific research stations or national facilities established in Antarctica, including both year-round and summer only operations [1]. Many stations have been operational for a long period, with 44 stations established prior to 1980, a further 35 established between 1980 and 2000, and at least 16 established since 2000. The most recent estimate of the disturbance footprint of human activity on ice-free land is >5,200,000 m^2^, which impacts more than half of all large coastal ice-free areas in Antarctica [2]. There are 62 stations situated in coastal areas [1] and many are very close to the coast for ease of access by ship.

Prior to the 1980’s little attention was given to the environmental impacts of station activities. Waste and rubbish were disposed of by dumping into landfill sites, onto sea ice, or into the ocean. From the 1980’s onwards environmental management practices improved greatly, largely due to the introduction and ratification of the Protocol on Environmental Protection to the Antarctic Treaty (known as the Madrid Protocol). For example, solid waste is now mostly exported from the continent. Historical practices have however, resulted in a legacy of environmental contamination [3–6]. As most stations are located in coastal areas, this can lead to contamination of local marine environments, with sources including sewage and wastewater discharges [7–10] oil spills [11–13], and waste disposal sites [14,15]. While pollution of marine environments is likely to occur at all coastal stations to varying degrees, it is not well documented and has only been reported for a few stations including McMurdo [16,17], Casey [18–20], Davis [7], and Rothera [9,21].

Our understanding of the processes that affect contamination of the Antarctic coastal marine environment is relatively limited. For example, it is not known how long existing contamination will persist or if natural processes will attenuate and/or distribute contaminants beyond existing contaminated areas. Similarly, our understanding of the impacts of such contamination on marine benthic ecosystems adjacent to stations, and the significance of such impacts in local and regional contexts is limited. To begin to address such issues it is important to ascertain the nature and extent of contamination of marine ecosystems around Antarctic stations. In this study we conduct a longitudinal analysis and summary of contaminants and sediment properties around Casey Station, collected over almost 20 years in a series of studies utilising a range of sampling and analytical methods.

### Casey station history

Australia’s Casey station was originally built between 1964 and 1969 on the Bailey Peninsula, as a replacement for Wilkes station 3 km to the north on the Clark Peninsula. Due to the building materials used, and its proximity to the sea, extensive corrosion of station buildings limited its life. A major rebuilding program of all of Australia’s Antarctic stations, including Casey, was undertaken in the 1980s and the old Casey Station was dismantled and removed from Antarctica. Building of the modern Casey station, 1km away from “Old Casey” was completed in 1988, with ongoing modifications, additional buildings and extensions happening periodically up to the present day. Casey station now consists of 18 permanent buildings and accommodates up to 120 people for short periods in summer, but has an average population of approximately 25 in winter and 90 people in summer.

**Fig 1:**
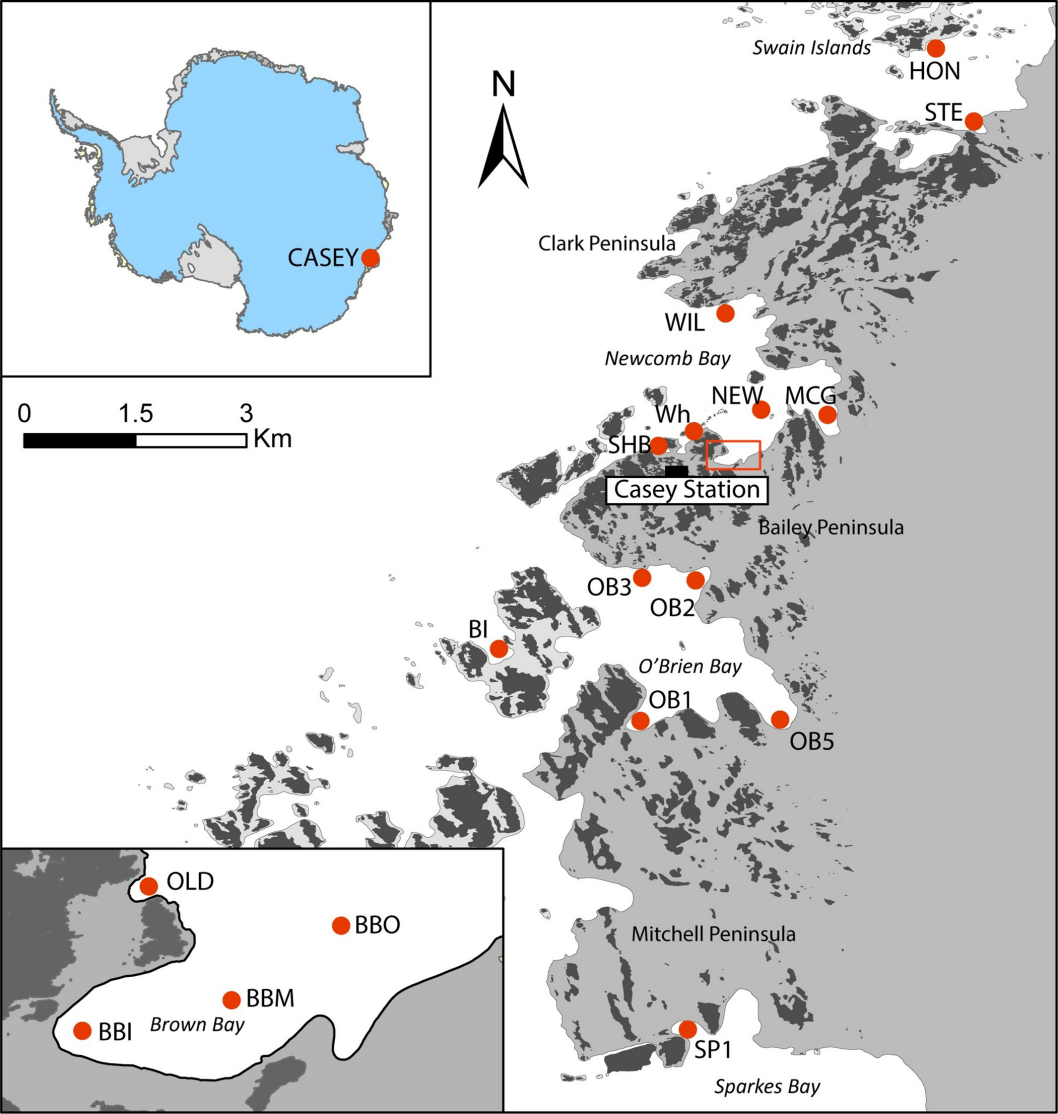
Map of sampling locations in the Windmill Islands around Casey Station. Upper inset shows location of Casey in east Antarctica. Lower inset shows Brown Bay as outlined in box on main Figure. HON = Honkala Island; STE = Stephenson Cove; WIL = Wilkes; NEW = Newcomb Island; MCG = McGrady Cove; Wh = Casey Wharf; SHB = Shannon Bay; OB1 to 5 = O’Brien Bay 1 to 5; BI = Beall Island; SP1 = Sparkes Bay 1; OLD = Old Casey; BBI = Brown Bay Inner; BBM = Brown Bay Middle; BBO = Brown Bay Outer.

Up until 1986, solid waste from Casey Station was disposed of in an above-ground waste disposal site at Thala Valley, on the foreshore of Brown Bay (Fig 1), within Newcomb Bay. Materials disposed of included ash, vehicle parts, batteries, empty oil drums and other metal, glass, plastics, paper, cardboard, wood, rope, clothing, construction materials, asbestos, cement, rubber, insulation batts and drums of unidentified waste chemical and waste oils. Active erosion by melt streams flowing through the site was estimated to annually mobilise 4 to 8 m^3^ of contaminated material into Brown Bay and the adjacent marine environment [22], and oil slicks were regularly observed leaching into the Bay during summer melt periods. A preliminary site assessment in 1994 [23] found high concentrations of metals and hydrocarbons leaching into Brown Bay. In recognition of Australia’s obligations under the Madrid Protocol, in the 1995/96 summer, 150 tonnes of waste (mainly large metal scraps and drums) were removed from the waste disposal site, and the remaining surface material pushed into a stockpile for future management. A short access road through the ice and snow was also added. This activity resulted in the dispersion of contaminants within the site and the mobilisation of contaminated material into the sea, which is likely to have increased contamination (metals and hydrocarbons) in Brown Bay [22].

The decision was made to remediate the Thala Valley waste disposal site in 2000, informed by extensive research into the most appropriate methods to use to prevent further environmental contamination and damage [14]. In 2003/04 1800 m^3^ of waste material and contaminated soil was removed for return to Australia for disposal. Meltwater trenches were constructed in the valley to minimise the volume of water flowing through the waste disposal site, and a holding pond on the shoreline of Brown Bay was used to contain leachate runoff from the site. An onsite custom designed water treatment plant separated particulates and removed dissolved metal contaminants before treated water was released into Brown Bay [24–27]. Total and leachable concentrations of metal contaminants in soil were assessed at Casey [28] and approximately 1000 m^3^ of waste and contaminated soil was returned to Australia where it was processed (by chemical fixation) prior to deep burial at a site approved for biosecurity hazardous waste. Approximately 800 m^3^ of waste was stockpiled at Casey Station and protected from meltwater by diversion channels, with leachate control including geofabric/membrane barriers and bunding. In 2011 this remaining stockpiled waste was returned to Australia for disposal at an authorised secure landfill site in Western Australia.

A comprehensive monitoring program was established prior to the clean-up operations to evaluate its effectiveness [14]. This included monitoring of short term effects during the clean-up operation [29], medium term effects in the 2 years following the clean-up [30] and long term effects and ecological recovery (to be reported in future).

The modern Casey station also included a wastewater treatment plant which provided rudimentary secondary treatment using a rotating biological contactor system. In peak periods when the station population exceeded 60 people, the working capacity of the plant was exceeded, and the system was routinely bypassed. Wastewater is discharged to the nearshore marine environment at Shannon Bay via an outfall positioned at the top of ice cliffs. Heated effluent has melted a hole in the ice down to bedrock, whereby the discharge makes its way into Shannon Bay. This wastewater treatment plant is currently being replaced with modern tertiary treatment plant using membrane filtration technology.

Research into the extent and ecological impacts of contamination in terrestrial and marine environments around Casey commenced in 1997. Assessment of contaminants in sediments in marine environments close to Casey Station has taken place at irregular intervals since 1997 as part of various survey and monitoring projects. Surveys of marine microbial, diatom, meiofaunal and macrobenthic communities and habitats found significant differences at impacted locations in comparison to controls location [18,31–36]. Experimental work demonstrated that these differences, including community composition, lower species richness and diversity, and dominance by species of polychaetes and crustaceans, were largely related to contamination in sediments [37–43].

### Aims

In this paper we examine long term changes and trends in contaminants in marine sediments at impacted and control locations including metals, hydrocarbons, nutrients and some persistent organic pollutants (POPs) such as polychlorinated biphenyls (PCBs) and polybrominated diphenyl ethers (PBDEs). We address the following questions:

○ How do sediment properties such as grain size and organic matter content vary?
○ How does contamination of marine sediments vary spatially in marine environments close to Casey station?
○ Have contaminant concentrations changed over time?
○ How did the clean-up of a former waste disposal site affect contaminant levels?
○ Is the wastewater outfall introducing contaminants?
○ How does marine contamination at Casey station compare to other Antarctic stations and other coastal sites on a global level?

## Methods

### Windmill Islands and casey station

The Windmill Islands is an area of low rocky islands and peninsulas in Vincennes Bay, East Antarctica (Fig 1). The shallow marine environment (< 30 m) around the many bays and islands that form the coastline consists of a highly heterogeneous mosaic of bedrock outcrops, boulders, cobbles and gravel interspersed with areas of soft sediments ranging from course sand to fine muds (Stark et al 2003a). Australia’s Casey station (66° 17’S, 110° 32’E) is located on the Bailey Peninsula at the southern edge of Newcomb Bay. Seven of the study locations are within Newcomb Bay. Samples were collected under ATEP/AMLR permits issued by the Australian Antarctic Division (AAS projects 2201, 2948, 4127, 4180).

### Site descriptions

Brown Bay, at the south-western corner of Newcomb Bay, is adjacent to Thala Valley, the location of a waste disposal site active from 1965 to 1986 (Fig 1). Brown Bay has a shallow, gently sloping bathymetry and was sampled at three areas, treated for statistical purposes as Locations; Inner (5–8 m water depth), Middle (12–15 m) and Outer (15–20 m). Brown Bay is generally free of sea ice for 1–2 months each year between January and March. A melt stream flows through Thala Valley in summer and drains into the bay. For many years this stream flowed through the Thala Valley waste disposal site, entraining rubbish, particulates and dissolved contaminants, and depositing them into the bay [22]. A variety of rubbish items are present on the sea floor in the bay.

Old Casey is a small embayment adjacent to a small gully that runs to the waters edge from the top of the ridge where the former Casey Station was located, which may have been a source of contaminants into the bay. Samples were taken in 5–8 m depth approximately 50 m from shore.

Wilkes station, now abandoned, is a former USA/Australian station on the northern side of Newcomb Bay on the Clark Peninsula, which included a waste disposal site approximately 100 m from the shore of Newcomb Bay (Fig 1). Summer melt water runs through waste disposal site to the nearshore marine environment, although there is no defined melt stream or permanent channels. Sampling was conducted approximately 30 m from the shoreline at 12–15 m depth.

Shannon Bay, adjacent to Casey station, is a small embayment bordered by ice cliffs (Fig 1). At the waterline, a steep slope with large boulders extends down to around 15 m depth, below which a relatively homogeneous muddy sand substrate extends to 25 m (Stark 2000). The Casey Station wastewater and sewage effluent is discharged from a pipe 30 m from the cliff edge, where there is a large melt hole, which extends to bedrock and thus provides a pathway for effluent into the bay.

Casey Wharf is on the southern shore of Newcomb Bay and is where all station cargo and refuelling operations are conducted, as well as being the location of a boat ramp. A large diesel fuel spill occurred from fuel storage behind the wharf in 1990 [23]. Boating operations disturb sediments around the wharf and there is some debris scattered around the wharf on the sea floor.

McGrady Cove is a small bay in the south-eastern corner of Newcomb Bay, bordered by steep rocky slopes and ice cliffs (Fig 1). Samples were taken from the sea floor on the northern side of the bay which consists of a gently sloping sediment covered bench that extends the length of the bay, out to approx. half way into the bay. No operational activities occur in McGrady Cove and it is considered a control location.

Newcomb Island (unofficial name) is a small rocky outcrop within Newcomb Bay, with relatively steep sides that drop down to mixed habitats of sediment and rocky reef at 15–30 m.

O’Brien Bay, on the southern side of the Bailey Peninsula (Fig 1), contains several small embayments bordered by a mix of gentle to steep rocky slopes and steep, high ice cliffs. No operational activities take place in O’Brien Bay but it has been the focus of multiple scientific studies. O’Brien Bay-1 slopes gently from the southern shore (5 m depth) to the outer edge of the bay (20–25 m), with a relatively flat sea floor of sediments interspersed with patches of rock, cobbles and large boulders. O’Brien Bay-2 and -3 are steeper sided with a series of submarine terraces, with a variable mosaic of habitat ranging from rock and gravel to cobbles and boulders interspersed with sediment patches. O’Brien Bay-5 has a moderate slope with terraces and benches descending to a relatively flat muddy bottom. O’Brien Bay-2 and -3 are generally ice-free for 3 to 4 months of the year between December and March. However, O’Brien Bay-1 and -5 generally have longer ice duration, with open water occurring less regularly, or not at all in some years and multi-year ice is estimated to be present for periods of 3 to 5 years.

Sparkes Bay-1 is a small embayment on the northern edge of Sparkes Bay, on the southern side of the Mitchell Peninsula, south of Casey station (Fig 1), well removed from station operations. Ice cliffs border the northern edge of the Sparkes-1 while the eastern edge is a gently sloping rocky valley. A gradually sloping shoreline descends to the sea floor at 5 to 10 m depth of mixed habitats of rocky reef, boulders and fine sediments. Sediments in Sparkes Bay-1 have naturally occurring high levels of some metals (Cd, Cu, Ni, Zn, Sb) in comparison to other control locations [18].

Beall Island is located to the southwest of the Bailey Peninsula, just outside the entrance of O’Brien Bay (Fig 1). Sampling was conducted in a small bay bounded by ice cliffs. The sloping rocky sides of the bay grade to a sea floor (20–30 m depth) of sandy sediment, gravel and boulders with patches of the large seaweed *Himantothallus grandifolius*. The site is commonly ice-free for 4–5 months between November and March due to being more exposed than other sites and can be temporarily ice free even in winter.

Honkala Island is in the Swain Island group to the north-east of Casey Station (Fig 1). Sampling was conducted on the south-eastern edge of the Island in 15 to 30 m water depth. It is semi- enclosed, well sheltered, with sea ice cover for 10 to 11 months of the year.

Stephenson Cove is a small enclosed bay at the north-eastern edge of the Clark Peninsula, to the north-east of Casey Station (Fig 1). A rocky sill spans the entrance to the bay, with a deeper basin within, resulting in the retention of hypersaline brine when sea ice forms which results in anoxic bottom waters. Dead fauna have been observed in this anoxic zone. Samples were taken well above this zone in sediments not affected by anoxia (no evidence of surface bacterial mats or dead fauna).

### Sampling history

Sediment samples were collected during summer field seasons between 1997 and 2015 (Table 1) either by divers with hand corers or a remotely operated sediment grab.

**Table 1:** Years of marine sediment sampling at locations around Casey station and the Windmill Islands, East Antarctica, between 1996 and 2015.

	96/97	97/98	98/99	05/06	06/07	14/15
Potentially Impacted Sites						
Brown Bay Inner	g	C	C	C	C	C
Brown Bay Middle	g	C	C	C	C	C
Brown Bay Outer		C	C			C
Wilkes		C	C	C		C
Casey Wharf						
Shannon Bay	g	C			C	C
Old Casey		C				
Control Sites						
O’Brien Bay 1		C		C	c	C
O’Brien Bay 2	g	C	C			C
O’Brien Bay 3	g	C				C
O’Brien Bay 5				C		
McGrady Cove				C	C	C
Sparkes Bay 1			C			
Beall Island	g					
Honkala Island					C	
Stephenson Cove					C	
Newcomb Island	g					

C = sediment core, g = sediment grab.

#### Sampling design

A nested hierarchical sampling design was used which incorporated multiple spatial scales, including: Locations (separated by several to 10’s of km); Sites within Locations (separated by approx. 100 m); and Plots within Sites (separated by approx. 10 m). Plots were generally 2–5 m in diameter and 2 to 4 replicate samples were taken inside this area. The number of sites within locations was generally 2, but up to 5 (at Shannon Bay in 2006); and the number of plots within sites was generally 2 but up to 3 in 2006.

In 1998 a survey was conducted in Brown Bay using a stratified design, with samples taken at each of nine sampling stations along three transects [35]. Transects started near the shoreline adjacent to the waste disposal site and ran parallel to each other with sampling stations at distances of: 50 m, 60 m, 70 m, 80 m, 100 m, 150 m, 200 m, 300 m and 400 m along the transects.

### Field sampling methods

#### Sediment grabs

A Van Veen sediment grab (20 x 25 cm– 0.05 m^2^ surface area sampled) was used to collect sediment samples [31] from a boat from 7 locations in February and March of 1997 (Table 1). The contents of the grab were placed into a plastic bag and frozen on station for transport to Australia for analysis. Prior to analysis the samples were defrosted and homogenised and sub-samples of sediment were collected for heavy metal, hydrocarbon, and POP analysis.

#### Sediment cores

Sediment was collected by divers using corers after 1997. Divers pushed a PVC core tube (5 cm diameter x 15 cm long) into the sediment to a depth of 10 cm and capped the bottom and top of the core before extraction. Cores were kept upright during return to the surface and transport to station. On station, cores were frozen at -20°C before either being extracted from the core tube and returned to Australia in a plastic ziplock bag (1997 to 2005/06) or frozen in the core tube (2006/07, 2014/15). In the 1998 survey of Brown Bay, one sample was also taken at each site for hydrocarbon analysis. An acid-washed glass jar was pushed into the sediment and on removal from the sediment was sealed underwater with an aluminium foil-lined lid.

#### Chemical and grain size analysis methods

Analyses were carried out on the top 5 cm section of sediment cores except for the grab samples (see above). For cores, either the top 0–5 cm section was removed from the frozen core, or it was sectioned into 1 cm intervals and a composite 0–5 cm sample was prepared by combining approximately equal portions by weight from the sections. These 0–5 cm samples were homogenised with a clean stainless-steel or plastic spatula, then subsampled for various analyses into a clean glass Petri dish. The analytical methods employed are summarised below and full details are included in the S1 File section and at https://data.aad.gov.au/metadata/AAS_4180_Marine-sediment-contaminants_Casey_1997-2015 with the data used in the study.

#### Grain size

For core samples taken prior to 2014, the outer 5 mm edge of the top 5 cm of the core was removed with a scalpel blade and the remainder dried at 45 ⁰C, then sieved through a 2mm sieve. The <2 mm fraction and the >2 mm fraction were separately weighed. A 5 g sample of the <2mm fraction was analysed with a Mastersizer 2000 Particle Size Analyser with Hydro 2000MU accessory at the Department of Physical Geography, Macquarie University, Sydney. Grain size analysis of sediment sampled in 2014/15 was performed at Geoscience Australia, Canberra ACT. A 50 g subsample of each core (0–5 cm composite) was dried at 40°C. The sediment was wet sieved mechanically to measure the general size distribution: gravel (>2 mm) / sand (2 mm– 63 μm) / mud (<63 μm). Precise determination of particle size by laser diffraction was done on a subsample of <2 mm material using a Mastersizer 3000 analyser fitted with a Hydro LV automated wet dispersion unit. The volume distribution data were used to calculate standard Wentworth size classes, ranging from clay (<2 μm) to very coarse sand (1.00–2.00 mm).

#### Total organic matter

Total organic matter (TOM, % of dry weight) was determined gravimetrically by loss on ignition (LOI) following the method described by [44]. Homogenised wet sediment (1–10 g) was dried at 105°C overnight in a porcelain crucible to determine the dry matter fraction (DMF). This was then ignited at 550°C for 4 h in a furnace to oxidise the organic material, cooled in a low humidity environment, and reweighed to measure the mass loss. For the 1998–99 data set, total organic carbon (TOC) in dry sediment was determined at AGAL, Pymble NSW with a Dohrmann combustion infrared TOC analyser using USEPA method 9060 [35].

#### Metals

Elements were extracted from sediments using dilute (1 M) hydrochloric acid, a commonly employed partial (selective) extraction method for identifying contaminated sediments [45,46]. This method broadly targets metals in labile sediment phases (e.g. carbonates, Fe and Mn oxides, sulphides, organics) where anthropogenic metals are most likely to reside. Dilute acid (1 M HCl) extraction data may also be correlated generally with bioavailability and hence potential ecotoxicity. Like all selective extraction methods, however, this is an operationally-defined procedure [47,48]. Consequently, the magnitude and reproducibility of the results obtained from the method, and its effectiveness in discriminating between contaminated and control sites, are dependent on the method parameters, including acid concentration, extraction temperature and time. Most of the data considered here involved extraction for 4 hours as recommended by [46], but the 1997, 1998, 1999 analyses used shorter periods (S1 Table) from 30 minutes to 1 hour. For most metals this was found not to have a significant effect on comparisons among locations, but for a few metals the longer extraction time resulted in higher concentrations including chromium, manganese, iron and nickel (S2 Table and S1 Fig in S2 File). Thus comparisons of pre-2000 data with 2014 for these metals were affected by an underestimation of pre-2000 levels, which was between two to three times larger for iron, chromium, nickel and manganese, but the effect was relatively small, and similar between impact and controls. This has been considered in interpretation of results.

Aside from variable extraction time, all extractions employed a 1:10 w/v (wet sediment) or 1:20 w/v (dry sediment) 1 M acid digest of a 2 to 5 g subsample of homogenised sediment at room temperature. Following centrifugation and/or filtration at 0.45 μm the extract was analysed by ICP-MS or ICP-AES. In all data sets, quality control was facilitated by extraction and analysis of two marine sediment certified reference materials (CRMs): MESS-2/3 and PACS-2 (National Research Council Canada, NRCC). Analytical confidence is also indicated, which is inferred from reporting limits, which changed over the duration of the study depending on instrument and laboratory (S1 Table).

#### Petroleum hydrocarbons and persistent organic pollutants

Analyses of total petroleum hydrocarbons (TPH) and the persistent organic pollutants polybrominated diphenyl ethers (PBDEs) and polychlorinated biphenyls (PCBs) in sediment samples were conducted by the Analytical Services Unit (ASU), Queen’s University, Kingston, Ontario, Canada. Analysis of TPH fractions C6-16, C16-34, C34-60 and TPH was performed as prescribed in [49,50]. Hydrocarbons were extracted from 5–10 g wet sediment with solvent and then, following concentration and clean-up, determined by gas chromatography with flame ionisation detection (GC-FID).

PBDEs and PCBs were extracted from 1–5 g of air-dried sediment with dichloromethane and concentrated by evaporation. Clean-up of extracts was performed by gel permeation chromatography followed by activated magnesium silicate. Analysis of the most common PBDE congeners was undertaken by gas chromatography with tandem mass spectroscopy (GC/MS/MS) while total PCBs were determined by gas chromatography with electron capture detection (GC-ECD).

#### Nutrients

Water-extractable nutrients (NOx, ammonia and orthophosphate) were measured in the 2014/15 sediment samples only. A 5 g wet subsample of the 0–1 cm core section was extracted with deionised water (1:5 w/v) for 1 h, centrifuged and the supernatant filtered through a 0.45 μm membrane. Flow injection analysis (FIA) of extracts was done at Analytical Service Tasmania (AST) using procedures based on standard APHA colorimetric methods.

### Statistical methods

#### Univariate and multivariate analysis

To test for differences in contaminants among and within sites, and to determine the contribution of different scales to variance estimates, we used permutational analysis of variance, PERMANOVA [51,52], based on a Euclidean distance similarity matrix [53] using the PRIMER program v7 [54]. This approach was used as it is well suited to handling unbalanced designs, with missing replicates or different levels of sampling (e.g. sites or plots). Planned comparisons were used to compare individual impacted sites with the group of control sites. Multivariate patterns of environmental variables were also analysed using Principal Component Analysis (PCA) and ordinations.

Figures displaying box-whisker plots represent all samples within a season at each location. The box extends from the 1^st^ to the 3^rd^ quartile, the line in the box is the median and the whiskers are the min and max values. Where outliers are present, the whisker on the appropriate side is drawn to 1.5 x the inter quartile range.

To assess the level of metal enrichment in sediment at impacted compared to background values (the average of control sites) the enrichment factor (EF) was calculated using the formula:

$$EF=(\frac{C_{m}}{C_{Al}})Sample/(\frac{C_{m}}{C_{Al}})Background$$

where C_m_ and C_Al_ are the concentrations of the trace metal (m) and aluminium. This gives a measure of metal enrichment normalized to Al, generally the major metal constituent of the clay minerals of the sediment matrix, as recommended in many studies [55–57]. This procedure is usually employed for total digests but in this investigation the aluminium concentration serves to normalise the partial extraction of geogenic metals from the matrix and the (typically) more complete extraction of anthropogenic metals.

### Evaluation of ecological risk

To evaluate the potential ecological significance of individual contaminants, the observed concentrations were compared to a range of sediment quality guidelines values (SQGVs) including those from Australia/New Zealand ANZECC/ARMCANZ Sediment Quality Guidelines [58]. The SQGV‐High values for metals are based on the effects‐range median (ERM) values [59] and for PCBs on the probable effect levels (PELs) [60]. Probable Effects Level (PEL) and/or the Threshold Effects Level (TEL) from the NOAA Screening Quick Reference Tables [61,62] were also used to assess risk at three levels: <TEL indicates that adverse effects are rare; from TEL to PEL indicates occasional adverse effects; and >PEL indicates that adverse effects are frequent [55]. For tin we compare observed concentrations to the Negligible Concentration (NC) from the Dutch National Environmental Quality Standards [63]. The Mean Enrichment Quotient (MEQ) was calculated for metals [64], to estimate normalized enrichment over a group of three common anthropogenic metals (copper, lead and zinc).

## Results

### Variation in sediment properties

**Fig 2:**
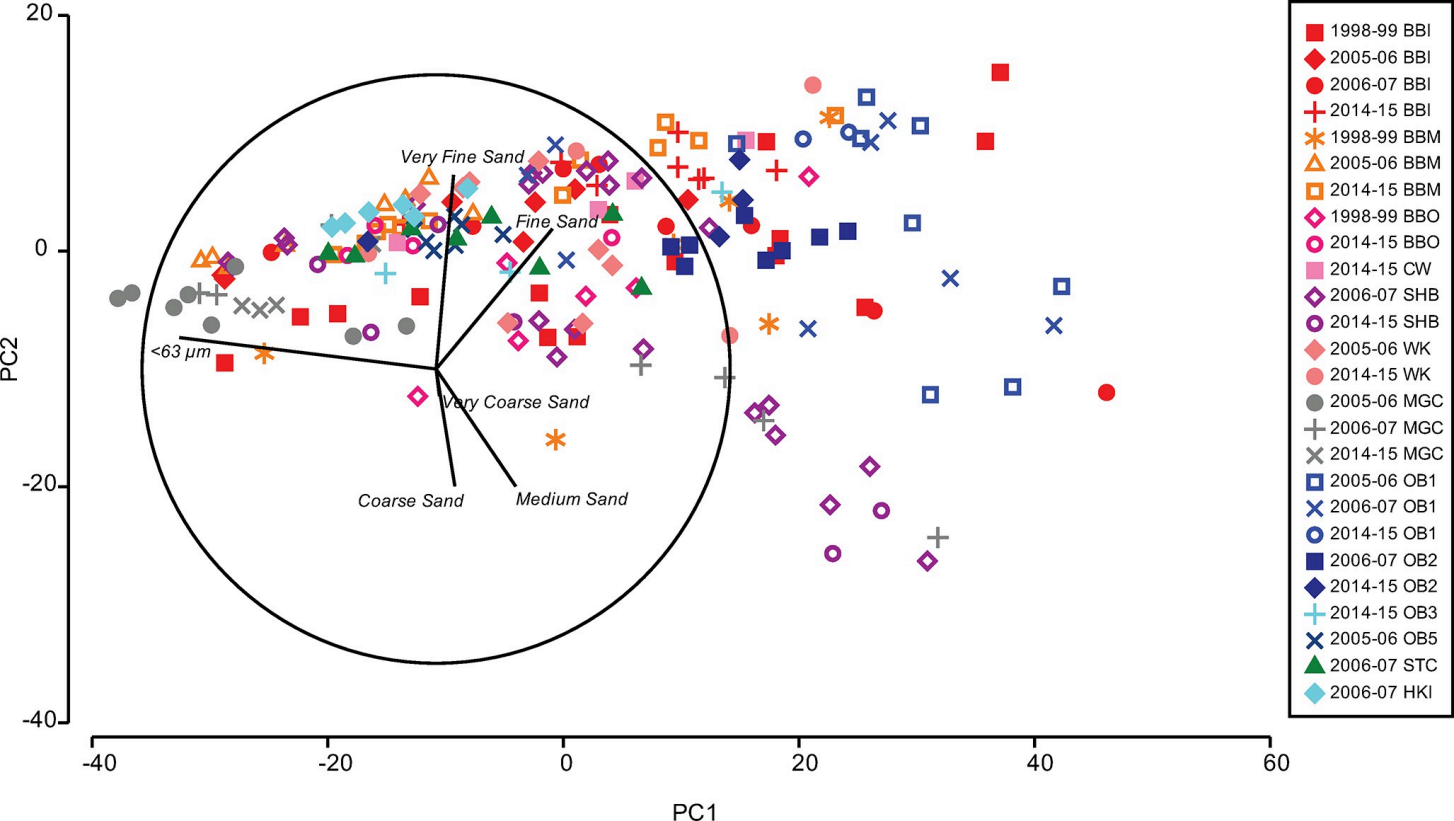
Spatial and temporal variation in sediment grain size. PCA ordination of % composition of grain size classes in sediments. PC1 and PC2 explain over 93% of total variation in grain size. Red to pink symbols represent potentially impacted locations and green, blue and grey represent control locations. A vector plot shows samples dominated by the <63 μm fraction on the left of the ordination with a gradient of increasing sand content correlated with PC1, but also a separation of samples with a gradient of medium and coarse sands to fine sands aligned with PC2.

Sediment properties, particularly grain size and organic matter content, can have a strong influence on contaminant levels in sediments [65,66]. We examined how sediment properties vary spatially and temporally in order to aid interpretation of differences in their contaminant loads. There was considerable spatial and temporal variation in grain size properties of sediments, among and within locations, as can be seen in the wide dispersion of samples in the multivariate PCA (Fig 2).

#### Spatial variation in sediment grain size and organic matter content

**Fig 3:**
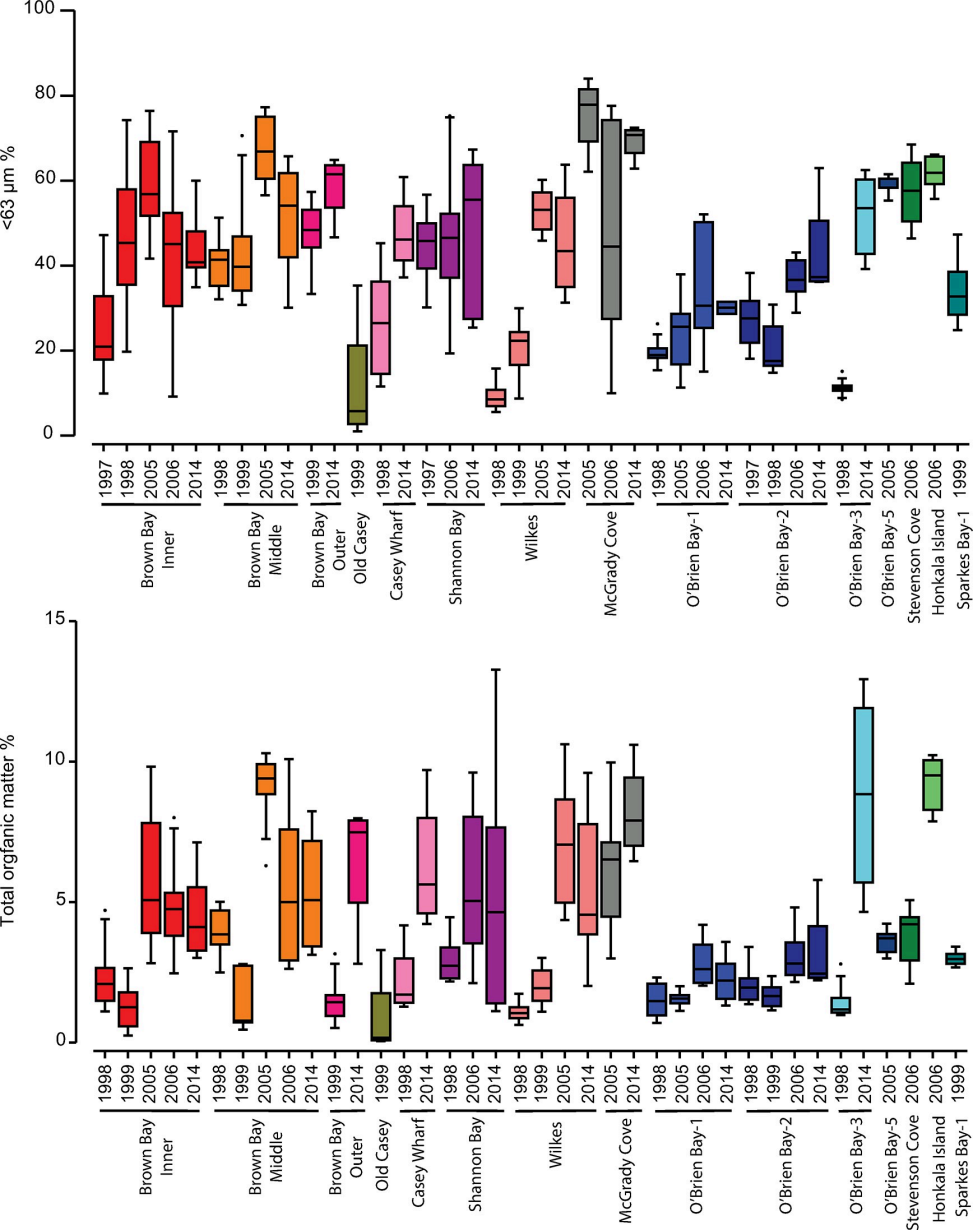
Spatial and temporal variation in marine sediments at Casey. Box and whisker plots showing: A) the percentage of <63 μm fraction and B) % of total organic matter in marine sediments at Casey station in the Windmill Islands, East Antarctica sampled between 1998 and 2014.

Analysis of spatial variation in grain size sediments was done by examining differences in % of different size classes of the sediment including: <63, 63–125, 125–250, 250–500, 500–1000 and 1000–2000 μm. This was done for samples of sediment from surveys in 1998; 2005/06, 2006/07 and 2014/15. Some locations (e.g. Brown Bay Inner, O’Brien Bay-1) have a particularly large degree of spatial variation in grain size. Four-factor multivariate analysis of grain size categories showed there was a strong difference among locations and an effect of time but that there was no location x year interaction (Table 2). There was however significant variation within locations between sites and plots and these were examined on a location by year basis (Table 3). Overall, the location and plot scales explained the most variation in grain size, followed by year of sampling (Table 2). However when the <63 fraction was examined separately it showed a very strong location x season interaction (differences among locations changing over time) (Table 2). Organic matter, in contrast, showed strong small scale variation at the plot scale, but there were also strong trends of differences among locations (Table 2). The locations of Brown Bay, Shannon Bay and McGrady Cove tended to have a higher proportion of <63 μm and higher TOM content than some of the control locations in O’Brien Bay (Fig 3). The control location of McGrady Cove in Newcomb Bay had among the highest proportion of <63 μm (Fig 3). O’Brien Bay locations showed a general trend of an increase in <63 μm component and also organic matter content, particularly at O’Brien-2 and -3 (Fig 3).

**Table 2:** Four factor PERMANOVA analysis of all grain sizes, <63 μm and total organic matter (TOM) in sediments.

	All grain size		<63 μm		TOM	
Source	P(perm)	% var	P(perm)	% var	P(perm)	% var
Location	0.01	23	0.1	10	0.006	16
Year	0.006	11	0.0001	30	0.0001	41
Location x Year	0.3	3	0.0001	28	0.03	8
Site(Loc x Year)	0.08	10	0.02	7	0.06	7
Plot(Site(Loc x Year))	0.0002	22	0.005	8	0.0001	19
Residual		31		17		10

**Table 3:** Spatial variation in grain size classes of marine sediments at Casey, by year of sampling.

Size class μm		P(permutation)			Estimates of components of variation (%)			
		Location	Site	Plot	Location	Site	Plot	Residual
<63	1997/98	**0.005**	**0.006**	0.73	61	14	0	25
<63	2005/06	**0.004**	0.46	**0.03**	81	0	8	11
<63	2006/07	**0.004**	0.17	0.11	54	9	9	27
<63	2014/15*	0.3	**0.03**	0.06	11	41	26	22
				**mean**	52	16	11	21
63–125	2005/06	**0.03**	0.96	**0.001**	31	0	44	25
63–125	2006/07	0.15	0.48	**0.001**	16	0	47	37
63–125	2014/15*	0.05	0.00	**0.200**	42	43	4	10
				mean	30	14	31	24
125–250	2005/06	**0.004**	0.10	0.46	72	7	0	21
125–250	2006/07	**0.01**	**0.02**	0.32	47	22	3	28
125–250	2014/15*	**0.14**	**0.19**	0.07	22	18	33	28
				mean	47	16	12	25
250–500	2005/06	**0.009**	0.72	**0.0002**	47	0	41	13
250–500	2006/07	0.45	0.55	**0.002**	0	0	55	44
250–500	2014/15*	0.50	0.01	**0.030**	0	61	28	11
				mean	16	20	41	23
500–1000	2005/06	0.10	0.85	**0.0002**	12	0	71	17
500–1000	2006/07	0.39	0.40	**0.03**	2	4	40	54
500–1000	2014/15*	0.40	0.00	**0.00**	3	87	8	0
				mean	6	30	39	24
TOM	1997/98	0.008	0.64	0.0009	48	0	29	23
TOM	2005/06	0.004	0.65	0.019	67	0	15	18
TOM	2006/07	0.196	0.09	0.0001	17	31	36	16
TOM	2014/15*	0.025	0.42	0.0001	33	3	55	9
				**mean**	**41**	**9**	**34**	**16**
**Degrees of Freedom**		Location	Site	Plot	Residual			
	1997/98	8	9	18	36			
	2005/06	5	6	12	23			
	2006/07	6	10	14	37			
	2014/15	9	12	19	10			

Results of 3 factor PERMANOVA analyses of grain size classes within different years, showing P values for each factor, and estimates of components of variation. Significant results in bold type. Degrees of freedom for each factor in each year of survey shown at bottom (varied with slight changes in replication).

* = test for plot in 2014/15 only for Brown Bay Inner and Middle.

Of the individual grain size fractions, the proportion of the fines (<63 μm) had the biggest differences between locations (Fig 3), with coarser fractions generally having less difference among locations (Table 3). For example, the location scale for <63 μm in sediments explained between 11 to 81% of the overall variation, with a mean of 52% (Table 3), whereas the location scale accounted for less variation for coarser fractions (Table 3). Significant variation in grain size and TOM also occurred at smaller scales, within locations (plot and site) and were generally greater between plot than between sites. Residual (random) variation was responsible for a large component of overall variation (Table 3), demonstrating the poorly sorted structure of Antarctic sediments, lack of sorting processes and the strong influence of stochastic processes on sediment grain size.

### Temporal variation in grain size and TOM

There were also strong differences in sediment properties between years at some locations (Figs 2 and 3). The <63 μm fraction showed a very strong location x year interaction (differences among locations changing over time) and a strong effect of year (Table 2). Total organic matter also showed very strong changes over years (41% of estimated total variation), but the location x year interaction was relatively small (Table 2). Some locations had large differences between years, particularly at the disturbed locations of Brown Bay Inner, Middle, Outer and Casey Wharf, but also at the control of O’Brien Bay-3 (Figs 2 and 3).

We further examined whether there had been a change in sediment properties over time, in particular pre and post clean-up of the waste disposal site in Thala Valley adjacent to Brown Bay (done in 2003/04), by comparing samples take prior to 2000 to samples taken in 2014 (Table 4). At Brown Bay Inner there were significant differences among years in <63 μm but there was no difference between pre-2000 and 2014. At Brown Bay Middle there was a significant increase in the <63 μm fraction in 2014 compared to pre-2000. At Brown Bay Outer there was no significant difference in the <63 μm fraction in 2014 compared to pre-2000. At Shannon Bay there was no significant difference in grain size over time, At Wilkes there was a significant increase in the proportion of <63 μm in sediment and TOM in 2014 compared to pre-2000 levels. At the Control locations there was some variation in grain size over time, but less than at impacted sites for TOM, with the exception of O’Brien Bay-3 where there was a large increase in TOM from 1998 levels (Table 4, Fig 3).

**Table 4:** Results of PERMANOVA planned comparisons of grain size and TOM in 2014/15 versus pre 2000.

Impacted Location	<63 μm	TOM	All GS
Brown Bay Inner	ns	0.002	ns
Brown Bay Middle	0.03	0.003	ns
Brown Bay Outer	ns	0.008	0.05
Casey Wharf	0.02	0.003	NA
Shannon Bay	ns	ns	ns^A^
Wilkes	0.0004	0.0003	ns^A^
Control locations			
McGrady Cove^A^	0.02	0.05	0.02
O’Brien Bay-1	0.02	0.02	ns^A^
O’Brien Bay-2	0.001	0.01	ns^A^
O’Brien Bay-3	0.01	0.007	0.007^B^

^A^ = No pre 2000 data and single factor analysis done for year.

### Variation in sediment contaminants

#### Metals

There were very significant differences in metal concentrations among locations (Tables 5 and 6) and in most cases variation within locations was relatively small, although there were some exceptions to this generalization within particular seasons (e.g. large plot effect for copper in 2006/07, Table 6). The location scale explained a greater amount of total spatial variation for metals that it did for grain size with an average 55% of variation attributable to this scale (Table 5). There were generally no significant differences in metals between sites within locations (< 6% variation on average) but there was significant variation at the plot scale within sites (approx. 17% of variation on average) (Tables 5 and 6). Variation in metals was similar to variation in grain size at the site scale, but less for plot scale, with similar residual variation (Tables 3, 5 and 6).

**Table 5:** Four factor PERMANOVA analysis of metals in sediments.

	Pb		Zn		Cu		Fe		Sn	
Source	P	% var	P	% var	P	% var	P	% var	P	% var
Location	0.0001	51	0.001	31	0.002	27	0.0001	31	0.0001	48
Year	0.003	3	0.0001	12	0.05	3	0.0001	11	0.5	0
Location x Year	0.0004	12	0.04	9	0.05	10	0.001	10	0.2	4
Site(Loc x Yr)	0.9	0	0.6	0	0.6	0	0.97	0	0.2	4
Plot(Site(Loc x Yr))	0.07	6	0.0001	27	0.0001	39	0.003	20	0.03	12
Residual		27		21		20		27		33

**Table 6:** Spatial variation in metal concentrations in sediments, by year, at Casey.

		P (permutation)			Estimates of components of variation (%)			
Variable	Year	Location	Site	Plot	Location	Site	Plot	Residual
Pb	1997/98	**0.0001**	0.95	**0.0004**	56	0	27	17
Pb	2005/06	**0.0006**	0.95	0.91	79	0	0	21
Pb	2006/07	**0.01**	0.10	0.14	70	14	4	12
Pb	2014/15*	**0.00**	**0.04**	0.99	68	3	0	29
Zn	1997/98	**0.007**	0.64	**0.003**	57	0	23	20
Zn	2005/06	**0.001**	0.61	0.21	87	0	2	10
Zn	2006/07	**0.01**	0.17	**0.02**	53	11	15	20
Zn	2014/15*	0.14	0.34	**0.01**	17	6	57	19
Cu	1997/98	**0.0001**	0.98	**0.001**	46	0	38	15
Cu	2005/06	**0.0002**	0.99	0.25	77	0	2	21
Cu	2006/07	**0.08**	0.30	**0.0002**	27	0	53	20
Cu	2014/15*	0.43	0.10	**0.01**	2	31	58	9
Fe	1997/98	**0.0003**	0.94	**0.001**	38	0	41	20
Fe	2005/06	**0.0004**	0.97	0.86	71	0	0	29
Fe	2006/07	**0.009**	**0.05**	0.74	60	15	0	25
Fe	2014/15*	**0.002**	0.80	0.55	53	0	0	47
Sn	1997/98	**0.0001**	0.97	**0.02**	49	0	24	27
Sn	2005/06	**0.004**	0.07	0.58	63	5	0	32
Sn	2006/07	**0.01**	0.11	0.39	60	14	1	24
Sn	2014/15*	**0.00**	**0.01**	0.77	58	16	0	26
			**Average (SE)%**		**55(5)**	**6(2)**	**17(5)**	**22(2)**
Degrees of freedom		Location	Site	Plot	Residual			
	1997/98	8	9	18	36			
	2005/06	5	6	12	23			
	2006/07	7	11	16	41			
	2014/15*	9	12	19	10			

Results 3 factor PERMANOVA analyses of metal concentrations within different years, with resulting estimates of components of variation. Significant results in bold type. Approximate degrees of freedom (df) for each factor in each year of survey shown at bottom (varied with slight changes in replication).

* = test for plot in 2014/15 only possible for Brown Bay Inner and Middle.

**Fig 4:**
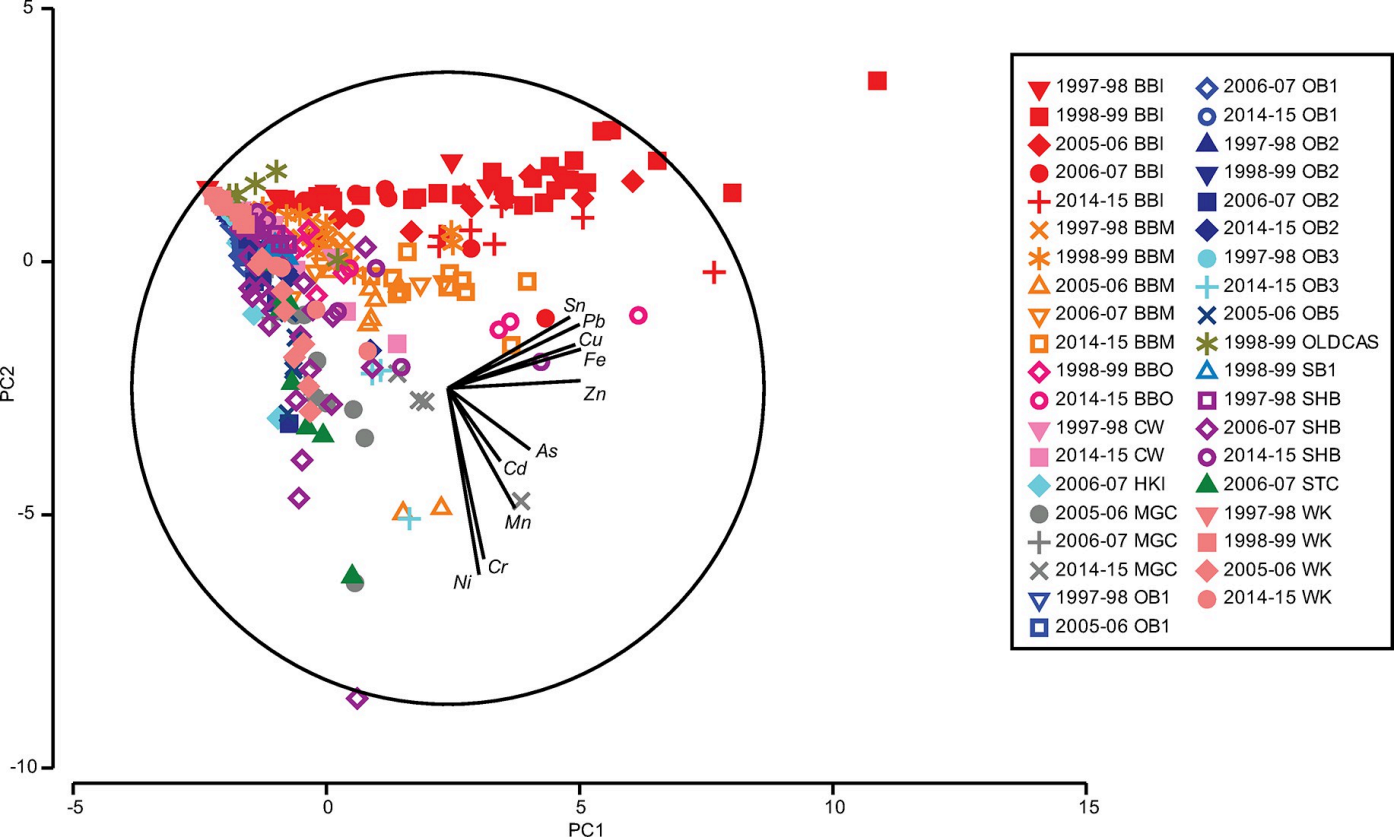
Spatial and temporal variation in metals in sediments at Casey. PCA ordination of metal concentrations in marine sediments at Casey station (normalised data). PC1 and PC2 explain 68% of variation in metals. Vector plot of correlations of individual metals with PC axes overlaid on ordination. BBI = Brown Bay Inner; BBM = Brown Bay Middle; BBO = Brown Bay Outer; CW = Casey Wharf; HKI–Honkala Island; MGC = McGrady Cove; OB1-3 = O’Brien Bay-1 to -3; OLDCAS = Old Casey; SB = Sparkes Bay; SHB = Shannon Bay; STC = Stevenson Cove; WK = Wilkes.

There was also strong temporal variation in metals among years of sampling, with the year factor explaining between 0- and 12% of variation, with a significant interaction between location and year (4 to 12% of variation, Table 5). At some locations, differences between years were obscured by high levels of spatial variability within locations, among sites or plots (Table 5). A PCA ordination of all metals demonstrates their strong spatial and temporal variation but also shows the distinct differences between control and impacted locations (Fig 4). The PC1 axis is correlated with concentrations of tin, lead, copper, iron and zinc and strongly differentiates Brown Bay Inner, Middle and Outer from all other locations, while the PC2 axis is correlated with concentrations of nickel, chromium and manganese and tends to reflect variation within locations for these metals (Fig 4).

### Impact versus control locations

**Fig 5:**
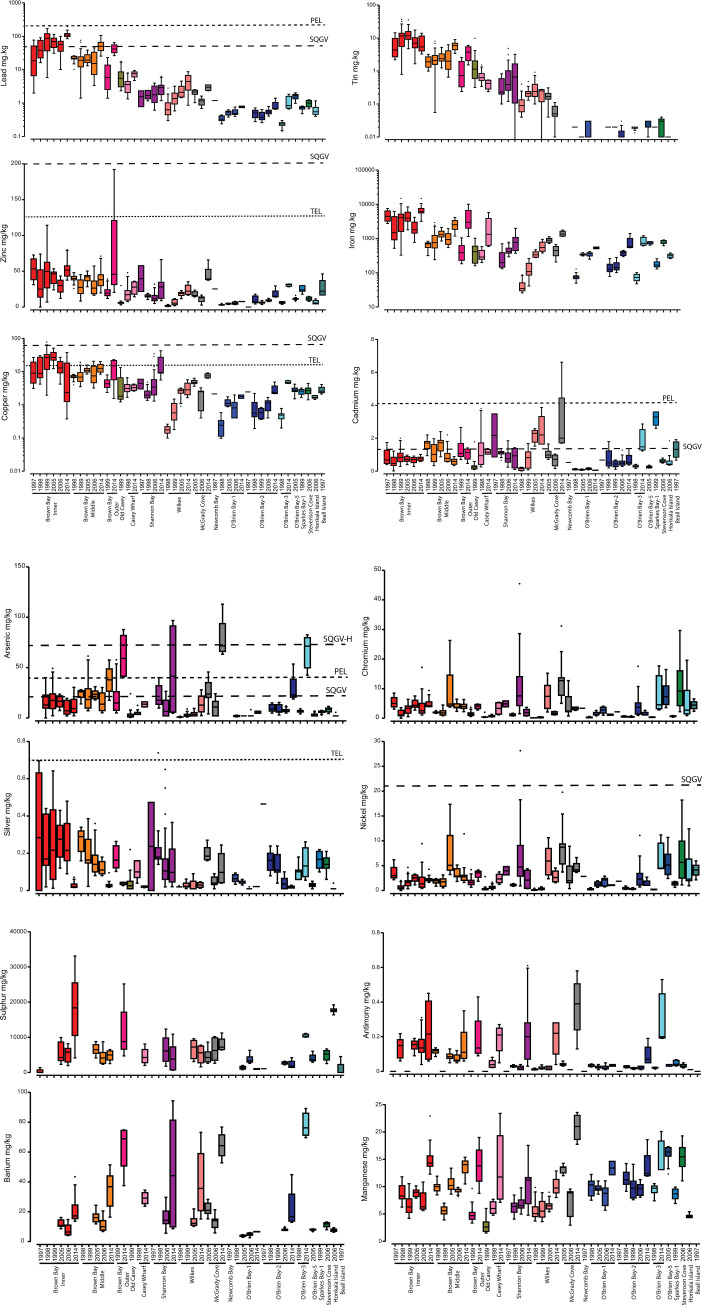
A and B: Spatial and temporal variation in metal and element concentrations in sediments at Casey. Box and whisker plots of metal concentrations: Each bar represents all samples in a particular year at a location. Dashed lines show various sediment quality guideline values: SQGV = ANZECC guideline trigger values; TEL = Threshold Effects Level; PEL = Probable Effects Level. Note log scale in some Figures. A) Lead, zinc, tin, iron, copper, cadmium; B) Arsenic, chromium, silver, nickel, sulfur, antimony, barium, manganese.

**Fig 6:**
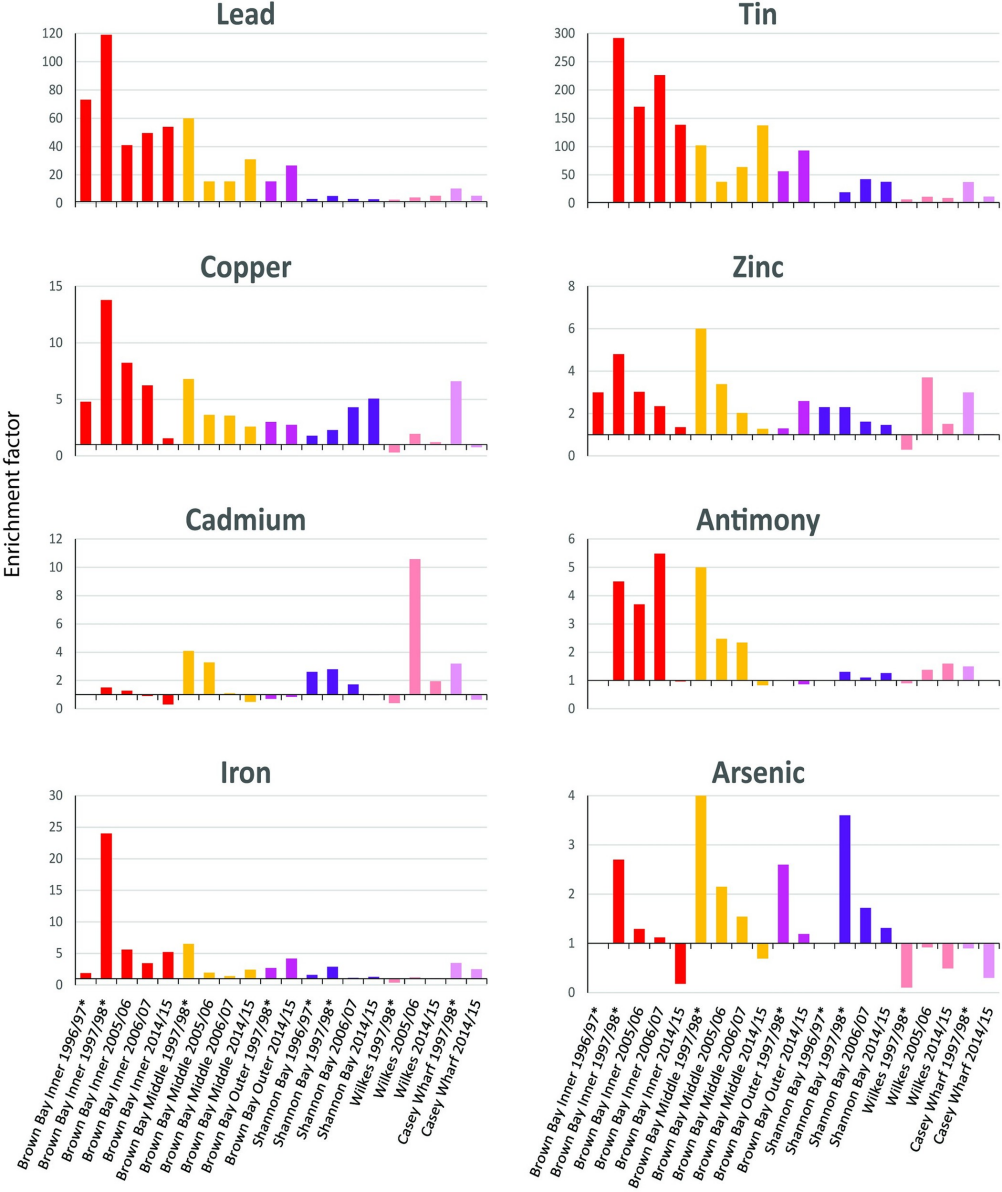
Normalized enrichment factors of metals in sediments at disturbed locations. Note that x-axis crosses y-axis at 1 and values below this are below the average of the control locations, while above 1 are enriched relative to controls.

Ratios of the concentration of metals at impacted compared to control sites demonstrate clear differences (Table 7). Concentrations of Pb, Sn, Zn, Fe, Cu in 2014 were significantly higher at Brown Bay Inner, Middle and Outer than the control locations, and were generally higher at Brown Bay Inner than at Brown Bay Middle and Outer, which were in turn higher than Casey Wharf, Shannon Bay and Wilkes respectively (Fig 5A, Table 7). Iron, lead and tin were higher at Casey Wharf than at controls. Copper; lead, tin and copper were higher at Shannon Bay than controls and lead, tin and cadmium were higher at Wilkes than controls. There were also trends of higher silver, antimony, arsenic and sulfur at impacted locations but were generally non-significant due to high levels of variability within locations (Fig 5B). We also examined enrichment factors, calculated by normalizing metal concentrations to aluminium at each site versus the controls, and these show a similar pattern, with cadmium, arsenic and antimony also seemingly enriched at some impacted locations, (Table 8, Fig 6).

**Table 7:** Impact versus controls: Ratio of metal concentration at each impacted site compared to controls in 2014/15.

	Sn	Fe	Pb	Zn	Cu	Sb	Mn
Brown Bay Inner	210***	7.9***	82***	2.1**	2.4*	1.2ns	1.0^ns^
Brown Bay Middle	165***	2.9***	37***	1.5	3.1***	0.7ns	0.9^ns^
Brown Bay Outer	107***	4.8**	31***	3.0*	3.2*	1.0^ns^	0.9^ns^
Casey Wharf	11.5***	2.5^ns^	5***	1.0^ns^	0.8^ns^	0.9^ns^	0.9^ns^
Shannon Bay	30**	1.0^ns^	2.1*	1.2^ns^	4**	1.2^ns^	0.7*
Wilkes	5.6**	0.7^ns^	3**	0.9^ns^	0.8^ns^	0.9^ns^	0.6*
	Ag	Al	As	Ba	Cd	Cr	Ni
Brown Bay Inner	0.4^ns^	1.5*	0.3**	0.5*	0.5^ns^	1.4^ns^	0.7^ns^
Brown Bay Middle	0.4^ns^	1.2^ns^	0.8^ns^	0.7^ns^	0.4^ns^	1.1^ns^	1.0^ns^
Brown Bay Outer	0.5^ns^	1.1^ns^	1.4^ns^	1.3^ns^	0.7^ns^	1.1^ns^	1.0^ns^
Casey Wharf	0.3^ns^	1.0^ns^	0.3^ns^	0.7^ns^	0.6^ns^	0.8^ns^	0.7^ns^
Shannon Bay	1.8^ns^	0.8^ns^	1.0^ns^	1.1^ns^	0.8^ns^	0.6^ns^	0.6^ns^
Wilkes	0.4^ns^	0.6^ns^	0.3^ns^	0.9^ns^	1.7*	0.5^ns^	0.8^ns^

Results of PERMANOVA analysis of planned comparisons of metal concentrations between impacted and all control sites in 2014/15. Significance indicated as: * = <0.05, ** = <0.01, *** = <0.001.

**Table 8:** Impact versus control: Enrichment factors (EF) in different years for metals at impacted sites at Casey station.

	Ag	As	Ba	Cd	Cr	Cu	Fe	Mn	Ni	Pb	Sb	Sn	V	Zn
Brown Bay Inner 96/97*	0.3			1.0	1.6	4.8	1.9		1.2	73.1				3.0
Brown Bay Inner 97/98*	2.2	2.7		1.5	4.0	13.8	24.0		2.1	119.0	4.5	292.0		4.8
Brown Bay Inner 05/06	2.4	1.3	0.9	1.3	0.5	8.2	5.6	0.6	0.4	41.0	3.7	170.2	2.9	3.0
Brown Bay Inner 06/07	3.5	1.1	0.6	0.9	0.6	6.3	3.5	0.6	0.4	49.6	5.5	226.3	2.0	2.3
Brown Bay Inner 14/15	0.3	0.2	0.3	0.3	0.9	1.6	5.2	0.6	0.4	54.0	1.0	138.5	1.9	1.4
Brown Bay Mid. 97/98*	2.5	4.0		4.1	4.9	6.8	6.5		5.6	60.0	5.0	102.0		6.0
Brown Bay Mid. 05/06	1.7	2.2	1.4	3.3	1.2	3.6	2.0	0.8	1.4	15.3	2.5	37.6	3.7	3.4
Brown Bay Mid. 06/07	1.4	1.5	0.8	1.1	0.4	3.6	1.4	0.6	0.5	15.3	2.3	63.9	2.0	2.0
Brown Bay Mid. 14/15	0.3	0.7	0.7	0.3	0.9	2.6	2.4	0.7	0.8	30.9	0.8	137.5	1.5	1.3
Brown Bay Out. 97/98*	1.2	2.6		0.7	2.5	3.0	2.7		2.1	15.3		56.4		1.3
Brown Bay Out. 14/15	0.4	1.2	1.3	0.6	1.0	2.8	4.2	0.8	0.8	26.6	0.9	93.1	1.7	2.6
Shannon Bay 96/97*	0.2			2.6	1.5	1.8	1.6		1.4	2.9				2.3
Shannon Bay 97/98*	2.3	3.6		2.8	2.8	2.3	2.9		3.4	5.0	1.3	18.9		2.3
Shannon Bay 06/07	3.3	1.7	2.0	1.7	1.9	4.3	1.1	0.8	1.9	2.9	1.1	42.3	1.3	1.6
Shannon Bay 14/15	2.3	1.3	1.4	0.7	0.7	5.1	1.3	0.8	0.8	2.7	1.3	37.8	0.9	1.5
Wilkes 97/98*	0.2	0.1		0.4	0.3	0.3	0.4		0.5	2.5	0.9	6.4		0.3
Wilkes 05/06	0.8	0.9	2.8	10.6	2.3	1.9	1.2	1.2	2.6	4.1	1.4	11.0	3.2	3.7
Wilkes 14/15	4.0	0.5	1.5	2.6	0.8	1.2	1.1	1.0	1.3	5.1	1.6	8.9	1.6	1.5
Casey Wharf 97/98*	1.0	0.9		3.2	1.9	6.6	3.5		2.2	10.3	1.5	37.2		3.0
Casey Wharf 14/15	0.3	0.3	0.7	0.8	0.9	0.8	2.5	0.8	0.7	5.1	1.0	11.5	1.3	1.0

EF calculated against average of controls in each season of sampling. Concentrations normalized to aluminium concentration at each location/year (linear correlation coefficient between Al and proportion of fines (<63 μm, <125 μm) for 2014/15 samples is 0.58 and 0.61, respectively). On average, the standard error of the EF for each element is 32% (range 26–38%).

* = not normalized to aluminium but just ratio of site to average of controls.

To determine whether long term changes in contaminants have occurred at impacted locations, including post remediation of Thala Valley (changes in Brown Bay) and also around other station areas including the sewage outfall and wharf, we made comparisons between sampling events prior to 2000 with samples taken in 2014 (Table 9). At Brown Bay Inner there was a general trend of an increase in lead from pre-2000 levels, while tin and copper show a trend of a peak in concentration around 2005/06 (Fig 5) and were less in 2014 than pre-2000. At Brown Bay Middle there were general trends of an increase in tin, copper, iron and lead from pre-2000 levels. At Brown Bay Outer most metals increased significantly from pre-2000 levels, and at Casey Wharf, iron and lead were higher in 2014 than pre-2000, although there are only two time points (1999 and 2014). At Shannon Bay, copper, lead and antimony were higher in 2014 than pre-2000. At Wilkes, cadmium, copper, iron, lead and zinc were higher in 2014 than pre-2000. The metals that increased at control locations from pre-2000 were barium, cadmium, copper, iron, manganese, lead, antimony and zinc (Table 9). However cadmium (Fig 5A) barium, manganese and antimony (Fig 5B) were not significantly different from impacted sites (which also increased) while copper, iron, lead and zinc were far lower than at impacted sites (Fig 5A).

**Table 9:** Change over time in metal concentrations. Ratios of metal concentrations between 2014/15 and pre-2000 at each location.

	Ag	As	Al^A^	Ba^A^	Cd	Cr	Cu
Brown Bay Inner	0.11**	0.8	1	2.2***	1.5*	1.4	0.4
Brown Bay Middle	0.14***	1.6	0.8	2.5***	0.4	1.5	1.7***
Brown Bay Outer	0.22**	3.3**			1.8*	2.7	3.0*
Casey Wharf	0.19**	2.6*			0.9	2.3	1
Shannon Bay	0.7	2.3	1.1	2.9**	0.5	0.6	5.1***
Wilkes	1.5	5.4**	1	2.9**	6.6***	4.3**	7.3***
McGrady Cove^A^ ©	1	4.4**	1.3	3.7***	5.6***	0.4	2.3***
O’Brien Bay-1 ©	0.5	4.2*	0.9	1.5**	2.1*	1.4	6.9*
O’Brien Bay-2 ©	0.08***	3.4**	1.2	2.9**	2.2*	1.5	4.1***
O’Brien Bay-3 ©	2.3	9.9*			11.5**	11.5*	10.2**
	Fe	Mn^A^	Ni	Pb	Sb	Sn	Zn
Brown Bay Inner	1.9**	1.9**	2.0	1.8**	2.4*	0.8	1.3
Brown Bay Middle	3.2***	1.4**	1.9	2.3***	1.4	2.4***	1.2
Brown Bay Outer	9.3***	2.8**	1.8	5.1**		3.3**	3.6**
Casey Wharf	6.5**	2.2*	2.1	2*	5.7**	0.6	1.4
Shannon Bay	1.6	1.5***	2.0	2.5*	8.4***	2.8	1.1
Wilkes	6.9***	1.5***	8.0**	3.7**	11.3**	1.2	5.9***
McGrady Cove^A^ ©	2.1***	2.1***	0.8	1.9***	14.5***	0.03*	3.1***
O’Brien Bay-1 ©	6.8*	1.5*	1.7	2.2*	1.5	2.5	2.3*
O’Brien Bay-2 ©	5.0*	1.5***	2.4	1.9**	4.4***	1.8	2.2**
O’Brien Bay-3 ©	11.4**	1.6**	18.2*	4.8**	16.3**	1.9	4.9**

Results of PERMANOVA analysis comparing differences between metals in 2014/15 season versus pre 2000. Significance indicated as: * = <0.05, ** = <0.01, *** = <0.001. ^A^ = No pre-2000 data and comparison done with 2005/2006. NA = no data for comparison. © = control locations

Patterns of individual metals and their analytical confidence can be summarised as follows:

Lead (confidence = high): Concentrations were significantly greater (2 to 82 times) at Brown Bay, Casey Wharf, Shannon Bay and Wilkes than the controls in 2014/15. Lead was between 2 and 5 times greater in 2014/15 than pre-2000 levels at Brown Bay, Casey Wharf, Shannon Bay and Wilkes (Table 9, Fig 5A). Lead also showed a slight increase at all controls, most likely as a consequence of analytical effects (difference in extraction time) although it was well below levels observed impacted locations. Normalized enrichment factors showed similar results, with EF ranging from 2.7 to 54 above background (Table 8, Fig 6). The lead EFs also show an increase at Brown Bay Inner, Middle and Outer from 2005 to 2014, but were well below pre-2000 levels, and an increase at Wilkes (Table 8, Fig 6). At Brown Bay in 2014 lead was above the SQGV and at the Inner site was also above the PEL (Fig 5).

Tin (confidence = medium): Patterns were very similar to lead, with levels between 6 and 210 times greater than controls in 2014/15 (Table 7). There was a significant increase at Brown Bay Middle and Outer in 2014/15 compared to pre 2000 (Fig 5A, Table 9). There was no significant change at controls. Tin showed changes in EFs, generally decreasing at Brown Bay Inner, increasing at Brown Bay Middle, Outer, and Shannon Bay (Table 8, Fig 6). Very few sediment quality guidelines exist for tin but all observed levels were well below the Dutch Negligible Concentration value (239 mg/kg) [63].

Copper (confidence = high): Concentrations were between 2.4 and 4 times greater at impacted sites (except for Casey Wharf and Wilkes) than controls in 2014/15 (Table 7). Levels in 2014/15 were higher than pre-2000 at most impacted sites with the exception of Brown Bay Inner and Casey Wharf (Table 9), however, EF’s decreased (Fig 6). There was a significant increase in copper at Brown Bay Inner between 1998 and 2005 followed by a decrease post-2005, however, there is large variability in copper concentrations in 14/15 (Fig 5). Significant increases at Shannon Bay and Wilkes from pre-2000 are clearer (Fig 5A, Tables 8 and 9). Concentrations of copper were below the SQGVs except in one sample at Brown Bay Inner, but were consistently above the TEL at Brown Bay and Shannon Bay (Fig 5A). There was a relatively small increase in Cu at control sites in in 05/06, 06/07 and 2014/15 compared to pre-2000.

Zinc (confidence = high): Concentrations in 2014/15 were significantly higher at the Brown Bay locations and at Wilkes than controls (1.5 to 3 times) (Table 7). Concentrations of zinc increased from pre-2000 levels only at Brown Bay Outer and Wilkes (Tables 8 and 9). Zinc also increased above pre-2000 levels at the control locations but by smaller amounts than at impacted locations (Fig 5A). EFs were above background at all impacted sites other than Casey Wharf and generally decreased from 2005/06 to 2014/15 (Table 8, Fig 6). Zinc was below sediment quality guidelines except at Brown Bay Outer, where some samples were above the TEL (Fig 5A).

Iron (confidence = high): Iron was significantly higher in 2014/15 at Brown Bay and Casey Wharf than control locations by between 2.5 and 8 times (Table 7). Iron concentrations generally increased significantly across all locations, including controls, from pre-2000 levels by between 2 and 11 times (Table 9). Iron EFs at impacted locations ranged from 1.1 to 5.6 with a large decrease at Brown Bay Inner from 1998 to 2014 (Table 8, Fig 6). Iron is not regarded as a toxic contaminant in marine sediments and there are no sediment quality guidelines. However, it is clearly elevated at impacted sites (Fig 5A) and is correlated with other metals owing to strong association in both the contaminant source materials and transport processes (e.g. metal adsorption onto Fe oxyhydroxide particles).

Cadmium (confidence = medium): Concentrations were not significantly different between impacted and control locations in 2014 (Table 7) but were generally elevated from pre-2000 levels, especially at Brown Bay Inner, Outer, Wilkes and controls (Fig 5A). EFs indicate greater levels than controls at Brown Bay Middle, Shannon Bay (EF 1.3 to 3.3) and especially Wilkes (EF 10.6) in 2005/06/07 followed by a significant decrease in 2014/15 (Table 8, Fig 6). Cadmium concentrations were above the SQGV at Brown Bay (all three sites), Shannon Bay, Casey Wharf and Wilkes and also, to a lesser degree, at some control sites. Concentrations of cadmium at controls were on average higher in 2014 than previous years, particularly at McGrady Cove and O’Brien-3, where they exceeded the SQGV in 2014 (Fig 5A).

Barium (confidence = high): Barium concentrations were not significantly different between impacted and control locations in 2014/15 except Brown Bay Inner where they were slightly lower than controls (Table 7). Barium increased from pre-2000 levels across all locations by a factor of 1.5 to 3.7 (Fig 5B, Table 9). No sediment quality guidelines exist for barium.

Manganese (confidence = high): Patterns of manganese were very similar to barium, with no significant differences between impacted and control locations in 2014/15 except for Shannon Bay and Wilkes which were slightly lower than controls (Table 7, Fig 5B). There was a general increase over time at all locations, ranging from 2 to 5.6 times higher in 2014 than pre-2000 levels (Table 9). No sediment quality guidelines exist for manganese.

Silver (confidence = low, near RL): In 2014/15 silver concentrations were not significantly different at impacted locations compared to controls (Table 7). There appears to be a significant decrease at Brown Bay and Casey Wharf in 2014/15 from pre-2000 levels (ratio 0.1 to 0.2) (Fig 5B, Table 9), although this should be interpreted with caution owing to the high uncertainty in the 2014/15 silver data. EFs indicated some enrichment of silver at Brown Bay and Shannon Bay in 2005–2007 and ranged from 1.4 to 3.5 (Table 8). Concentrations were below SQGVs but in a few samples exceeded the TEL (Fig 5B).

Antimony (confidence = low, near RL): There were no significant differences in concentrations of antimony between control and impacted locations in 2014/15 (Table 7), but EFs show strong enrichment at Brown Bay which appears to have decreased by 2014 (Fig 6). However there was a trend of an increase in antimony concentrations compared to pre-2000 at most locations (Fig 5B, Tables 8 and 9). As for silver, however, this may be an analytical artefact, especially for the controls, because of higher RLs for the 2014/15 data. EFs ranging from 1.3 to 5.5 show enrichment of antimony at Brown Bay, Shannon Bay and Wilkes (Table 8, Fig 6), but all levels were below the SQGV (2.0 mg/kg).

Arsenic (confidence = medium), chromium and nickel (confidence = high) were elements that showed no clear patterns in concentration across locations or time (Fig 5B). Arsenic showed some evidence of enrichment at Brown and Shannon Bays, with EFs ranging up to 4 (Table 8, Fig 6), but decreasing over time at Brown Bay. Concentrations were above the SQGV at most impacted sites but also above the PEL and the SQGV-high value at Brown Bay Outer and Shannon Bay (Fig 5B). The arsenic SQGV-high value was also exceeded at control sites in 2014/15 including McGrady Cove and O’Brien Bay-3 (Fig 5B).

Chromium was generally not enriched except in Shannon Bay and Wilkes samples from 2005–2007 (Table 8) and in all cases was well below the SQGV (80 mg/kg) (Fig 5B). Nickel also showed little consistent evidence of enrichment (Table 8) and, with the exception of one sample at Shannon Bay, was also below the SQGV (Fig 5B).

#### Hydrocarbons

**Fig 7:**
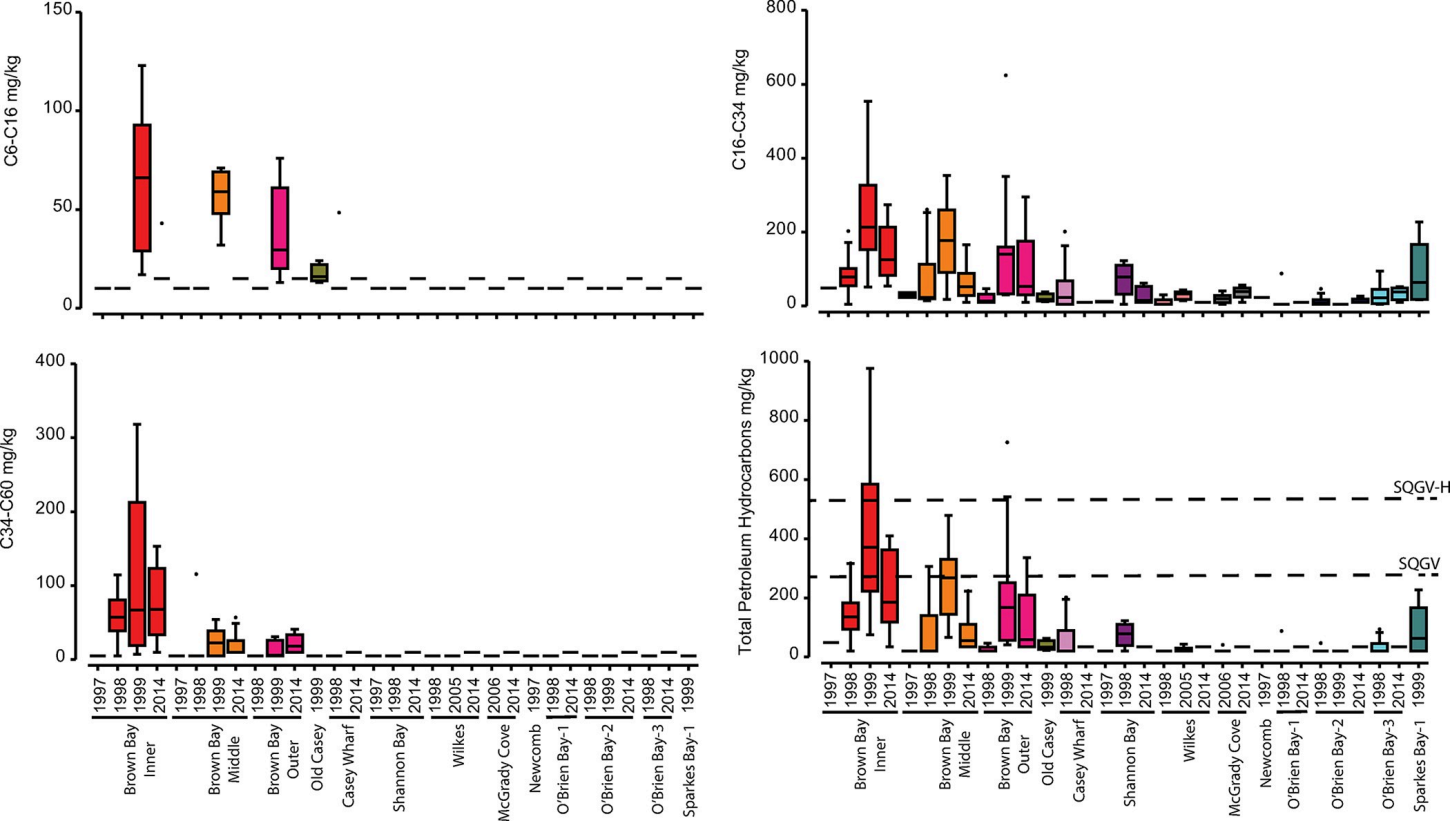
Spatial and temporal variation in hydrocarbon concentrations in sediments at Casey. Box and whisker plots of hydrocarbon concentrations: Each bar represents all samples in a particular year at a location. Dashed lines show various sediment quality guideline values: SQGV = ANZECC guideline trigger values; TEL = Threshold Effects Level PEL = Probable Effects Level.

Light hydrocarbons in the C6-16 range were only detected at Brown Bay, Old Casey and the Wharf, and heavy hydrocarbons in the C34-50 range were only detected at Brown Bay Inner, Middle and Outer. The most widespread hydrocarbon contamination was found for the C16-C34 range (Fig 7). TPH concentrations were significantly above SQGVs at Brown Bay and above the SQGV-high value at Brown Bay Inner in 1999 (Fig 7). Hydrocarbon concentrations were extremely variable within locations as seen in both the large residual variation and plot scale contributing up to 67% of variation in one survey (Table 10). The location scale contributed between 11 and 48% of total variation, with a mean of 30%, while variation at the site scale was small but was large at the plot scale, averaging 24% (Table 10), further demonstrating the patchy nature of hydrocarbon contamination. TPH concentrations were significantly greater than controls in 1998 and 2014 at Brown Bay Inner and Middle, and at Brown Bay Outer in 2014; and at Casey Wharf and Shannon Bay in 1998 but not in 2014 (Table 11).

**Table 10:** Spatial variation in hydrocarbons in sediments at Casey station.

		P(permutation)			Estimates of components of variation (%)			
Hydrocarbons		Location	Site	Plot	Location	Site	Plot	Residual
TPH	1997/98	0.3	0.4	**0.0006**	11	4	65	20
TPH	1998/99	**0.03**	0.06	NA	44	15	NA	42
TPH	2014/15*	**0.02**	0.2	0.9	26	3	0	71
C6-16	1998/99	**0.04**	**0.003**	NA	48	23	NA	29
C16-34	1997/98	0.5	0.4	**0.0007**	0	8	67	25
C16-34	1998/99	**0.02**	0.1	NA	40	12	NA	48
C16-34	2014/15*	**0.01**	0.6	0.7	34	0	0	66
C34-50	1997/98	**0.05**	0.9	0.2	44	0	11	45
C34-50	1998/99	**0.05**	0.2	NA	32	11	NA	57
C34-50	2014/15*	**0.02**	**0.007**	0.99	24	8	0	69
				mean	30	8	24	47
Degrees of freedom for tests at different scales					df	df	df	df
				1997/98	7	7	15	22
				1998/99	6	9		34
				2014/15*	9	12	19	10

Results of 2 or 3 factor PERMANOVA analyses of hydrocarbon fractions and total petroleum hydrocarbons (TPH) concentrations in different years, with resulting estimates of components of spatial variation. Significant results in bold type. Approximate degrees of freedom (df) for each factor in each year of survey shown at bottom.

* = test for plot in 2014/15 only possible for Brown Bay Inner and Middle.

**Table 11:** Comparison of TPH concentrations between impacted and control locations in 1998 and 2014.

	P(perm)	
Year	**1998**	**2014**
Location	**0.04**	**0.004**
Brown Bay Inner vs controls	**0.0001**	**0.0002**
Brown Bay Middle vs controls	**0.04**	**0.005**
Brown Bay Outer vs controls	0.8	**0.04**
Casey Wharf vs controls	**0.05**	NS
Shannon Bay vs controls	**0.003**	NS
Wilkes vs controls	NA	NS

Results of one factor PERMANOVA with planed comparisons of impacted vs control locations for TPH concentrations.

Temporal variation in hydrocarbons was only examined at Brown Bay (Inner, Middle and Outer) as at other sites there were either insufficient sampling times to make a comparison or, as in the case of most controls sites, hydrocarbon levels were at or below reporting limits. For the Brown Bay sites there were two or three time points prior to 2000 and only one post 2000, in 2014/15. There was no significant difference in TPH concentrations between pre and post 2000 measurements at Brown Bay. There is an apparent trend, however, of a peak in concentrations in 1999, with lower average concentrations in 2014 (Fig 7).

### Persistent organic pollutants (POPs)

Most of the variation in POPS was associated with the residual (Table 12A), indicating a large degree of variation among replicates, with Location accounting for between 14 to 61% for PCBs but only 3 to 5% for PBDEs. The site factor did not contribute to overall variation for PCBs, however, replication was limited (small numbers of samples were analysed due to cost) with no tests at the plot level. POPs were highly variable at small spatial scales, varying by 2–3 orders of magnitude within some disturbed sites, particularly PCBs as seen in the very large residual variation (Table 12A).

**Table 12:** Spatial variation in persistent organic pollutants in sediments at Casey.

**A)**		P(permutation)			Estimates of components of variation (%)					
	Year	Location	Site		Location		Site		Residual	
PCB	1997/98	0.14	0.50		14		2		84	
PCB	2014/15	0.002	0.92		61		0		39	
PBDEs	1997/98	0.19	0.65		5		0		95	
PBDEs	2014/15	0.25	0.78		3		0		97	
Degrees of freedom for different scales					df		df		df	
		PCB 1998			7			7		17
		PCB 2014			9			12		23
		PBDE 1998			7			7		15
		PBDE 2014			9			12		13
B)				PCBs		PBDEs				
Over all years				df	P(perm)	df				P(perm)
Year				4	0.1	4				0.5
Location				11	0.0001	11				0.6
Brown Bay Inner vs controls				1	**0.0001**	1				**0.06**
Brown Bay Middle vs controls				1	**0.04**	1				0.8
Brown Bay Outer vs controls				1	**0.0002**	1				0.06
Casey Wharf vs controls				1	**0.0006**	1				**0.0002**
Shannon Bay vs controls				1	0.7	1				0.1
Wilkes vs controls				1	**0.02**	1				0.9
Year x Location				10	0.01	9				0.5
Residual				76		64				
Total				101		88				
2014 only				df	P(perm)	df				P(perm)
Location				9	**0.0004**	9				0.5
Brown Bay Inner vs controls				1	**0.0001**	1				**0.0009**
Brown Bay Middle vs controls				1	**0.0002**	1				0.6
Brown Bay Outer vs controls				1	**0.009**	1				0.2
Casey Wharf vs controls				1	**0.008**	1				**0.001**
Shannon Bay vs controls				1	0.1	1				**0.002**
Wilkes vs controls				1	0.3	1				0.6
Residual				35		25				
Total				44		34				

A) Results of 2 factor PERMANOVA analyses of PCB and PBDE concentrations within different years, with resulting estimates of components of spatial variation. B) Planned comparisons of impacted versus grouped control locations; Significant results in bold type.

**Fig 8:**
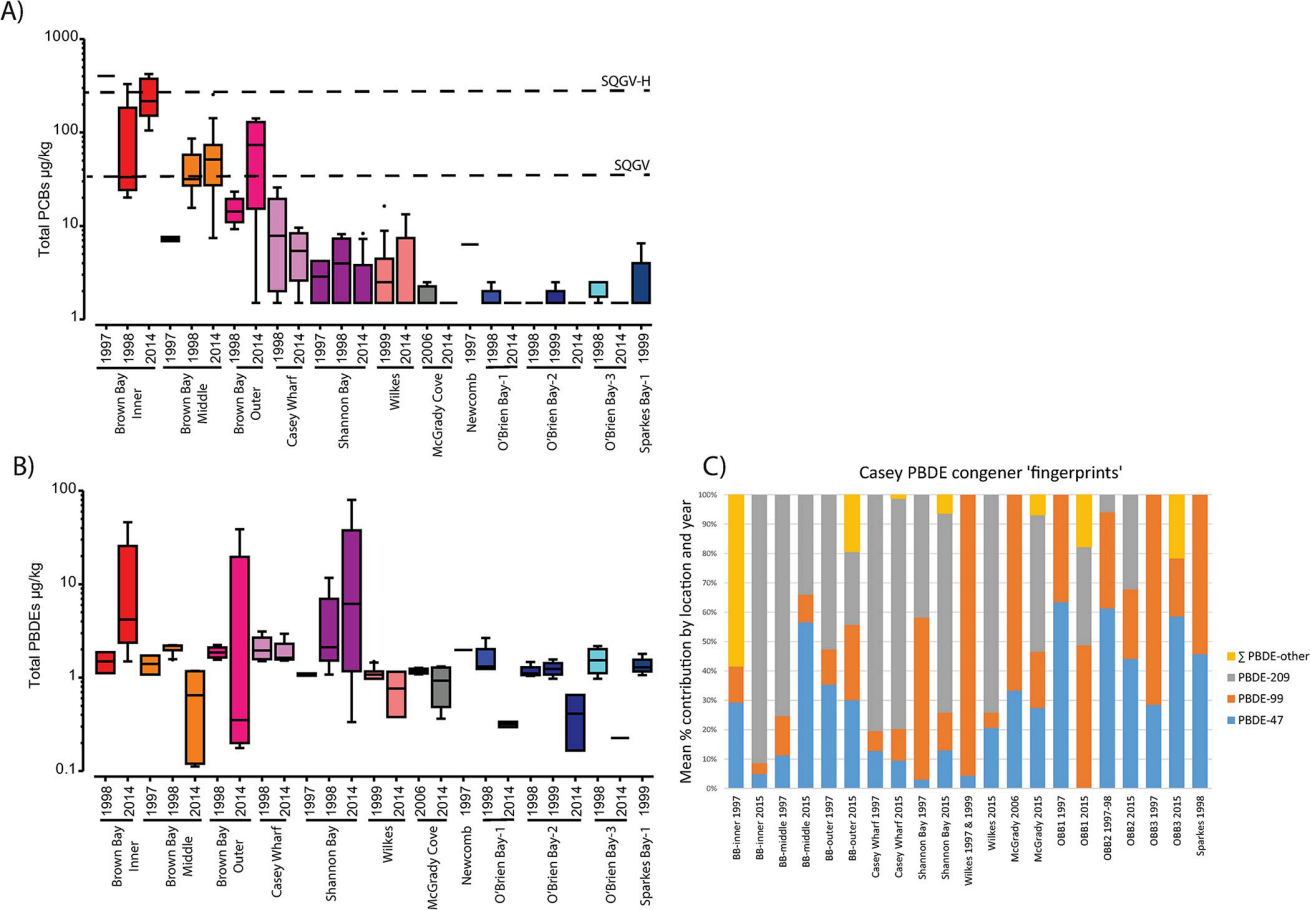
Spatial and temporal variation in persistent organic pollutants in marine sediments at Casey. Box and whisker plots of POP concentration for: A) PCBs and B) PBDEs (note log scale on Y axes), where each bar represents all samples in a particular year at a location. Dashed lines show various sediment quality guideline values: SQGV = ANZECC guideline trigger values; TEL = Threshold Effects Level PEL = Probable Effects Level; C) Average PBDE congener composition at each time at each location.

*PCBs*. There was very clear evidence of PCB contamination in Brown Bay, particularly at the Inner site, with levels persistently high between 1997 and 2014 and no evidence of a decrease, but a possible increase at the Inner and Outer sites (Fig 8A). There was also evidence of PCB contamination at Casey Wharf, Shannon Bay and Wilkes, with PCBs detected in most samples at consistently higher concentrations than the controls, which typically had levels below reporting limits. Total PCBs were significantly greater than controls at Brown Bay (all 3 sites), Casey Wharf and Wilkes but there were no significant differences among years (Table 12B). PCBs exceeded the SQGV at Brown Bay and the SQGV-high level at Brown Bay Inner (Fig 8A).

*PBDEs*. There was clear evidence of PBDE contamination in sediments from Brown Bay (Inner and Outer) as well as at Shannon Bay (Fig 8B). Concentrations were significantly greater than controls at Brown Bay Inner, Outer and Casey Wharf over all seasons (Table 12) and appear to have increased at these locations between 1998 and 2014. Some of the highest levels of PBDEs were observed at Shannon Bay, however, large residual variation between replicates meant that it was not significantly different from controls considered over all years. When 2014 was tested separately, however, Shannon Bay was significantly greater than controls (Table 12). Analysis of the congener profile at each location showed that at disturbed sites PBDE-209 was the dominant congener (Fig 8C), followed by PBDE-47 and PBDE-99, which is consistent with many ecotoxicological and site assessment studies as they are often the main congeners found [67]. Less common congeners present in two or more samples included PBDE-100, -154, -184, -206 and -207. (Fig 8C). There are no SQGVs for PBDEs.

#### Nutrients

**Fig 9:**
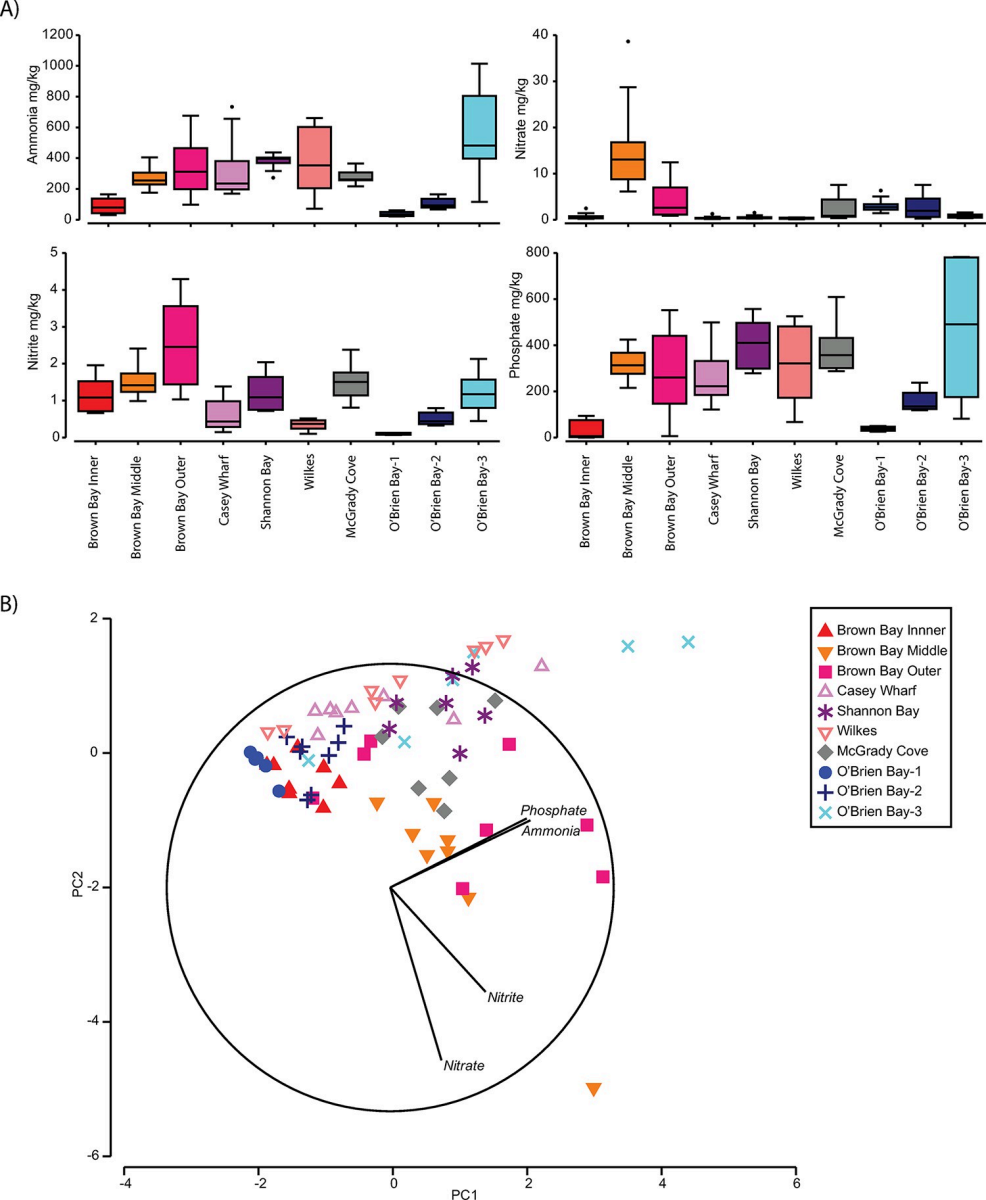
Spatial and temporal variation in nutrients in marine sediments at Casey. A) Box and whisker plots of nutrient concentrations in marine sediments at Casey station in 2014/15, w here each bar represents all samples; B) PCA ordination of nutrient concentrations (normalized data). PC1 And PC2 explain approx. 82% of variation in nutrients.

Most variation in nutrients was at the Location scale (44%), with the Plot scale also contributing 21% and residual variation also high (Table 13A). Nutrient concentrations were significantly greater at Brown Bay Inner, Middle and Outer than at controls (Table 13B, Fig 9A). In particular nitrate and nitrites were higher than at all other locations (Table 13B, Fig 9A). PCA analysis indicated there was also a gradient of phosphate and ammonia concentration (correlated with PC1 axis) but this did not differentiate control and impacted locations, which showed some differentiation on PC2 which was correlated with nitrite and nitrate (Fig 9B).

**Table 13:** Spatial variation in nutrients in sediments at Casey.

A)	P(permutation)			
	Location	Site	Plot	Residual
2014/15	**0.0005**	0.2	**0.0002**	
Est. components of variation (%)	44	7	21	27
B)	df	P(perm)	P(MC)	
Location	9	**0.0001**	**0.0001**	
Brown Bay Inner vs controls	1	**0.02**	**0.02**	
Brown Bay Middle vs controls	1	**0.0003**	**0.0001**	
Brown Bay Outer vs controls	1	**0.003**	**0.003**	
Casey Wharf vs controls	1	0.5	0.5	
Shannon Bay vs controls	1	0.1	0.1	
Wilkes vs controls	1	0.1	0.1	
Residual	64			
Total	73			

A) Results of 3-factor multivariate PERMANOVA analysis of nutrients in sediments (ammonia, nitrate, nitrite, phosphate), with estimates of components of variation for each scale; B) results of planned comparisons of impacted sites against control sites from 1 factor PEMANOVA. Significant results in bold.

## Discussion

There is a clear signal of anthropogenic contamination in the marine environment around Casey station, some of which results from legacy issues and past practices, but some of which may be ongoing. At disturbed sites, multiple pollutants are present in marine sediments at levels that are up to several orders of magnitude greater than background values at control locations. Several patterns of contamination were apparent: Brown Bay (adjacent to former waste disposal site) was contaminated with pollutants at levels higher than observed anywhere else at Casey. Shannon Bay (site of wastewater outfall) and the Casey Wharf also had consistently higher concentrations of contaminants than controls, while Wilkes (adjacent to an older waste disposal site) had lower levels and most contaminants were close to background values.

### Brown Bay and the remediation of Thala Valley

The remediation of the Thala Valley waste disposal site in 2003/04 created a disturbance to the terrestrial source of contaminants and led to a short-term pulse of contamination entering Brown bay in the 2003/04 summer [29], resulting in an increase in sediment metal concentrations [30]. In the two years post clean-up (2005 and 2006), there was a trend of greater concentrations of metals in sediments at Brown Bay Inner compared to other seasons, particularly for lead, tin, copper and iron. Subsequent to 2006, there was a further increase in enrichment factors for lead and tin at Brown Bay Middle and/or Outer in 2014 suggesting there may still be some input into the bay from terrestrial sources.

In contrast, there is evidence of a decline in enrichment factor of some metal contaminants post remediation (copper, zinc, arsenic, cadmium and silver), with lower concentrations and enrichment factors at Brown Bay Inner and Middle in 2014 compared to earlier surveys, possibly reflecting a reduction in source inputs. In some instances, there is a contrast between observed concentrations and enrichment factors, with increased concentrations at Brown Bay (e.g. copper) but a decrease in the normalized enrichment factor, due to corresponding increases in concentrations at control locations. Alternatively some metals in the sediment may have become more (or less) available in the 1 M HCl digest owing to changes in sediment redox conditions, affecting metal speciation [68]. This may be a consequence of increased anoxic conditions in the sediment because these observations were coupled with higher measurements of sulfur concentration at Brown Bay Inner, a location characterised by negative Eh potentials [68]. The importance of oxidation-reduction status and sulfidic minerals in determining metal availability in the benthic environment (and lability in sediment extraction procedures) has been investigated extensively [69,70], including sediments collected from the Brown Bay location [68,71]. Sulfur was not measured routinely but there is some evidence it has increased over time at Brown Bay Inner (Fig 5).

The measured decrease in hydrocarbons in Brown Bay Inner in 2014/15 points to the likelihood that some biodegradation of these contaminants has occurred. Given the history of Brown Bay, and the former waste disposal site on its shore being a source of hydrocarbons since the 1980s, sediment in this location is likely to have an enhanced community of hydrocarbon degrading bacteria [72]. Degradation rates, however, have been found to be slow in Antarctic marine sediments [73,74] and thus this contamination is likely to persist well into the future.

There was an increase in contaminant concentrations at Brown Bay Outer from pre-2000 to 2014, suggesting an offshore movement of sediment bound contaminants from the Inner to Outer sites. Anthropogenic source metals such as lead, tin, iron, copper and zinc increased at the Middle and Outer sites post the 2003/04 clean up. We hypothesise that this is due to resuspension and lateral transport of contaminated sediments within Brown Bay. Sediment resuspension can also promote oxidation processes within anoxic sediment and enhance the mobility of metals associated with sulfides [45,70]. Sediment redistribution could occur through several mechanisms including ice berg scour, melt stream flow into the bay and tidal currents. Iceberg scour is highly likely, as small ice bergs have been observed in Brown Bay in late summer when the sea ice breaks out. In some years these are frozen into the sea ice over winter and may have regular contact with the sea bed via tidal motion, lifting and settling the iceberg onto the sea floor and disturbing sediments. Alternatively, melt stream flow through the Thala Valley site into Brown Bay in summer may be causing sediment resuspension as well as transporting additional contaminants into the bay. A plume of fresh water flowing over seawater could result in greater deposition of sediment at Brown Bay Outer than the Inner and Middle locations. Contaminants may also come from other station sources and could be transported into the bay via the road between the station and the wharf, which is cleared of ice and snow annually and induces a large meltwater flow down through Thala Valley. Warming temperatures in the region [75] would enhance these meltwater processes, however there is no data on runoff, meltwater quantities or sedimentation rates in Brown Bay.

Persistent organic pollutants were also found in Brown Bay. The PCB contamination in Brown Bay is undoubtedly from the former Thala Valley waste disposal site, as it was noted to contain many items which would have contained PCBs [14,23]. PCBs are legacy contaminants derived from items and materials produced before the 1979 ban on PCB manufacture (USA Toxic Substances Control Act). Products containing PCBs disposed of at the site include: electrical equipment such as voltage regulators, switches, transformers, capacitors and electromagnets; oil used in motors and hydraulic systems; fluorescent light ballasts; thermal insulation material including fiberglass, felt, foam and cork; adhesives and tapes; oil-based paint; caulking and plastics. PBDEs were also one to two orders of magnitude higher at Brown Bay than at control sites. The main source of these organic contaminants is also likely to be materials disposed of at the Thala Valley site.

Other pollutants found in Brown Bay, particularly the Middle site, include significantly higher concentrations of nitrates and nitrites than other locations. Polycyclic aromatic hydrocarbons (PAHs) were not been measured in this study, but are likely to be present, as material in the waste disposal site was routinely burnt [23], which may have produced PAHs.

### Casey wastewater/sewage outfall (Shannon Bay)

The Casey wastewater outfall is a source of contamination into Shannon Bay. Enrichment factors show contamination by copper, zinc, cadmium, arsenic, silver, lead and tin. There were significant increases in concentrations of copper and lead in 2014 compared to pre-2000 levels, which were 4 and 2 times greater than at controls in 2014, respectively. Many of the water and wastewater pipes on Casey Station are made of copper and this may be the main source of this element into Shannon Bay. Lead may be coming from solder used to join copper pipes, as the construction of the station predates the banning of lead solder in plumbing applications in Australia in 1989 [76]. PBDE levels also increased in Shannon Bay between 1997/98 and 2014. Wastewater is a known source of PBDEs into Antarctic marine environments [7,20]. Sewage is also a source of nutrients, but although levels of some nutrients are relatively high at Shannon Bay (ammonia, phosphate), they are not significantly different from the controls, due to a large degree of variation between control locations, in particular, anomalously high values at O’Brien Bay-3 in 2014. If this data is excluded from comparison of control and impact locations, there is a much more significant difference between them in 2014. There is a trend of phosphate accumulation in sediments adjacent to the outfall, but further sampling is needed to test this. Sediments were not tested for evidence of other sewage derived pollution such as human enteric bacteria, viruses or antibiotic resistance mechanisms, but these are known to occur at other similar wastewater outfalls in Antarctica [10,77] as well as other emerging contaminants such as found in personal care products [78].

### Wilkes (abandoned station and waste disposal site)

There appears to have been an increase from pre-2000 levels in metal concentrations (cadmium, copper, iron, zinc, lead, arsenic) in sediments offshore from the waste disposal site of the former Wilkes station. Enrichment factors indicate well above background values for a range of metals including lead, tin, cadmium and zinc, with greater enrichment in 2014/15 compared to 2005/06. The proportions of fines and organic matter in the sediment were also greater in the years that metal levels were higher, suggesting a change in sediment properties may have contributed to this. There may have been an increase in the deposition of fines and organic matter into the marine environment adjacent to the Wilkes site, which introduced sediment-bound contaminants, however there are no data on runoff or snowmelt flowing from the land to sea in this location. Hotter summer temperatures [75] would increase meltwater runoff, potentially increasing input of dissolved contaminants and contaminated fines into the sea from the Wilkes waste disposal site. The presence of PCBs significantly above background levels is further evidence that Wilkes station is a source of contamination into the marine environment.

### Casey wharf

Enrichment factors demonstrate there is significant contamination by iron, lead, and tin at the Casey Wharf, while concentrations of iron, and to a lesser extent zinc, tin, lead and arsenic, increased in sediments over time. Metal items are visible on the seabed around the wharf, most likely coming from boating and cargo transfer activities, and may have contributed to an increase in metal concentrations in sediments. The wharf area is the site of intensive cargo and vehicle activity, and marine sediments have been observed to be disturbed by cargo barges. It is also exposed to occasional swell and waves, another source of sediment disturbance, resuspension and redistribution. Warming [75] and increased meltwater transport of contaminants, for example from the adjacent road, may also have contributed to contaminant increases. PBDE and PCB levels are also higher in wharf sediments than at controls. The wharf was the site of a large spill of diesel fuel in 1990 [23] and elevated levels of TPH were detected in 1998, but not in 2014, suggesting that residual contamination may have degraded or been diluted.

### Increase in metals at control locations

The concentration of arsenic, manganese, barium and iron increased in sediments across all sites including controls over the study period. Manganese and barium had similar patterns of change at impacted sites and controls, whereas iron, a major contaminant, increased to a greater degree at impacted sites than controls. Concentrations of Ba and Mn are 2 to 5 times higher in 2014/15 control samples compared to data obtained in 2005–2007. This difference may be in part due to spatial heterogeneity within locations, as shown in this study and [68]. However, it is consistent with an increase measured over the same period for aluminium and other elements such as rubidium (and in controls only, copper and zinc) associated with terrigenous sediment in the Windmill Islands [79]. One mechanism responsible for this increase may be an increase in meltwater flow carrying this material from the land into the near shore marine environment.

Another possible mechanism contributing to this observation is an increased level of primary production in coastal waters around Casey. Metals are important components of phytoplankton, where they are required for numerous physiological processes such as electron transport in photosynthesis and respiration (iron) and oxidation of water during photosynthesis (manganese) [80]. Common metals within metalloproteins in phytoplankton include (in order of abundance): iron, zinc, manganese, nickel, copper, cobalt, cadmium and molybdenum [80]. Environmental changes that could result in increased primary production include less sea ice cover, less cloud cover, and increased meltwater runoff (containing iron). If there was an increase in primary production, some of this material may have ended up in local marine sediments and contributed to the increase in the concentration of manganese and other metals in marine sediments.

Barium is a significant component of the regional terrigenous material and is correlated highly with the proportion of fines (<63 μm) in marine sediment samples (correlation coefficients for all 2014/15 samples: Ba 0.76, Al 0.58, Mn 0.61). Barium has increased in sediments by a greater degree than aluminium, rubidium, copper and zinc (~3.5 times), suggesting the increase is not primarily from terrestrial sources. Barium is not a component of metalloproteins, but it is associated with primary productivity. Dissolved barium can be concentrated from the water column by marine phytoplankton including diatoms, dinoflagellates and foraminifera and these organisms are thought to be potentially important vectors for the vertical transport of barium in the oceans [81]. Bacterial decomposition of dead phytoplankton in the water column leads to precipitation of barite, and barium has been used as a paleo-proxy for phytoplankton primary productivity in the ocean [82]. While best understood as a process of the open ocean, it may also play a role in the barium cycle in more shallow benthic environments [83], including near-shore Antarctic waters where seasonal phytoplankton blooms coupled with hypoxic conditions on the seabed, as observed by [84], could potentially result in barite precipitation.

### Ecological risk and comparison to other regions

To understand the potential ecological significance of the contamination at Casey we used a range of indices to assess and compare the level of contamination and estimate the potential ecological risk. We also compared concentrations of pollutants observed at Casey to other regions of the world in the World Harbours Project [56,85], an international collaboration examining the health of some major urbanised waterways around the world.

The Mean Enrichment Quotient (MEQ, based on Cu, Pb, Zn) values for Casey impacted sites indicated that environmental enrichment or modification for metals ranged from high (>5) (Brown Bay Inner, Middle and Outer) to moderate/slight at Shannon Bay and Wilkes, with slight enrichment at Casey Wharf (Table 14). Brown Bay has similar MEQ levels (highly modified, MEQ >10) to some heavily modified estuaries in Australia including the Derwent Estuary and Sydney Harbour; and to Rio de Janeiro. Metals at Casey also exceed the lower SQGVs, and in some instances exceed the PEL and TEL levels, and for arsenic they exceed the SQGV-high value. On the basis of metals alone Brown Bay poses a moderate level of ecological risk as measured by the MERMQ index, while Shannon Bay, Wilkes and Casey Wharf pose minimal to slight levels of ecological of risk (Table 14). Other contaminants were not able to be incorporated into an ecological risk index, but would potentially serve to increase the risk of negative ecological effects as some exceed sediment quality guidelines.

**Table 14:** Magnitude of anthropogenic change (MAC) and ecological risk posed by sedimentary contaminants.

	Magnitude of anthropogenic change (MAC)^a^			Ecological risk posed by sedimentary metals (ERA)^b^	
	MEQ All 12 metals (EF avg	MEQ for Cu, Pb, Zn EF avg	MEQ rating (Cu, Pb, Zn)	MERMQ (Cu, Pb, Zn)	MERMQ risk (Cu, Pb, Zn)
Brown Bay Inner 2005/06	20.0	17.4	high	0.35	slight
Brown Bay Inner 2006/07	25.2	19.4	high	0.26	slight
Brown Bay Inner 2014/15	17.1	19.0	high	0.51	moderate
Brown Bay Middle 2005/06	6.5	7.5	high	0.19	slight
Brown Bay Middle 2006/07	8.0	7.0	high	0.15	slight
Brown Bay Middle 2014/15	15.0	11.6	high	0.28	slight
Brown Bay Outer 2014/15	11.3	10.7	high	0.35	slight
Shannon Bay 2006/07	5.4	2.9	slight	0.05	minimal
Shannon Bay 2014/15	4.7	3.1	moderate	0.11	slight
Wilkes 2005/06	3.6	3.2	moderate	0.06	minimal
Wilkes 2014/15	2.5	2.6	slight	0.08	minimal
Casey Wharf 2014/15	2.2	2.3	slight	0.10	slight

Mean Enrichment Quotients (MEQs) calculated from Enrichment Factors (EFs) for marine sediments at impacted sites at Casey Station. All 12 metals includes: Ag, As, Ba, Cd, Cr, Cu, Fe, Mn, Ni, Pb, Sb, Sn, V, Zn.

^a^ MEQ < 1.5—not enriched; 1.5–3.0—slightly enriched; 3.0–5.0 –moderately enriched; > 5.0—highly enriched.

^b^ MERMQ < 0.1—minimal risk; 0.1–0.5—slight risk; 0.5–1.5 –moderate risk; > 1.5 high risk.

In comparison to other Antarctic stations, concentrations of metals in sediments at Casey impacted sites are much higher than those observed at Australia’s Davis station [7] but are broadly similar to those observed at impacted areas at the USA’s McMurdo Station [6,13]. Lead levels at Brown Bay are similar and in some cases higher than the severely contaminated Winter Quarters Bay, whereas most other metals (Cu, Zn, Cd) are slightly lower than at McMurdo [6]. Metal levels around Casey’s outfall are lower than those observed around the McMurdo outfall [6], which services a much larger population. Concentrations of PCBs recorded at Brown Bay exceed the SQGVs and are similar to those recorded in Winter Quarters Bay at McMurdo [86], most likely due to the similar nature of the sources. Winter Quarters Bay was once the location of a waste disposal site as well as workshops and still has a dock [15], all of which could have contributed PCBs and other contaminants into the bay. Levels of PCBs at the Casey outfall and wharf, however, are lower than those recorded at the equivalent McMurdo locations (although Casey wharf is not far below McMurdo wharf [86]), whereas levels at background sites are similar. Concentrations of hydrocarbons (TPH) in Brown Bay exceed the SQGVs but are generally lower than those observed in Winter Quarters Bay [87], although levels at the Casey outfall are higher than recorded at McMurdo’s outfall [6].

Higher concentrations of PBDEs were recorded at Casey in comparison to Davis Station, particularly at Casey wastewater outfall and Brown Bay Inner, however background levels are broadly similar (Table 15). PBDE levels at the Casey outfall are similar to those observed at the USA’s McMurdo Station outfall [17], with Brown Bay similar to the heavily contaminated Winter Quarters Bay at McMurdo (Table 15). PBDE levels at Casey, however, are roughly an order of magnitude lower than those found in developed or industrialised coastal areas in other regions of the world such as the Scheldt estuary in the Netherlands or San Francisco Bay (USA) (Table 15).

**Table 15:** Comparison of PBDE concentrations in marine sediments in Antarctica and other global regions.

	Year of study	n	∑PBDEs ug/kg (dry weight basis)	∑PBDEs ug/kg (TOC basis)	Ref.
**Casey** (This study)					
Casey Outfall (Shannon Bay)	1997–2014	12	0.3 to 80 (12.6)	2 to 3376 (526)	
Brown Bay Inner	1998–2014	6	1 to 46 (9.8)	5 to 1112 (233)	
Brown Bay Middle	1997–2014	11	0.1 to 2.2 (1.4)	2 to 32 (18)	
Brown Bay Outer	1998–2014	8	0.2 to 39 (6)	3 to 486 (84)	
Casey Wharf	1998–2014	4	1.5 to 3.1 (2)	16 to 134 (46)	
McGrady Cove	2006, 2014	7	0.3 to 1.3 (1.0)	1 to 15 (7)	
Wilkes	1999, 2014	10	0.4 to 1.5 (1.04)	2 to 29 (9)	
O’Brien Bay-1	1998–2014	6	0.3 to 2.7 (1.2)	10 to 63 (24)	
O’Brien Bay-2	1998–2014	10	0.17 to 1.5 (1.06)	3 to 26 (11)	
O’Brien Bay-3	1998–2014	6	0.23 to 2.2 (0.89)	2 to 31 (11)	
Sparkes Bay	1999	4	1.06 to 1.79 (1.36)	2 to 15 (7)	
**Davis**					**[7]**
Davis Outfall	2010	4	0.23 to 0.66 (0.49)	22 to 93 (50)	
Davis background	2010	5	ND to 0.27 (0.09)	ND to 25 (6.4)	
**McMurdo**					**[17]**
McMurdo outfall	2003	1		3910	
McMurdo Outfall South	2003	1		760	
Winter Quarters Bay Inner	2003	1		1420	
Winter Quarters Bay Middle	2003	1		499	
Hutt Point	2003	1		1820	
Jetty	2003	1		43.6	
Cinder Cones	2003	1		162	
Turtle Rock	2003	1		ND	
Cape Armitage	2003	1		ND	
Explorers Cove	2003	1		ND	
Cape Bernacchi	2003	1		ND	
**King George Island**					
Great Wall Bay	2009/2010	1		0.24	[88]
**Other regions**					
San Francisco Bay, USA	2007	48	ND—212 (9.63)		[89]
Korean coast	2004	25	0.22–494 (27.8)		[90]
Scheldt estuary, Netherlands	2001	3	240–1650		[91]
Tokyo Bay, Japan	2002	9	1 to 90		[92]
Pearl River estuary, China	2004	10	1 to 190		[93]

Numbers in brackets are averages; ND = not detected.

### Ecological effects

The ecological effects of this contamination are not fully understood and will be explored in future work. Past research has demonstrated clear differences in assemblages of meiobenthic and macrobenthic fauna in marine sediments between these contaminated locations and control areas around Casey [18,33,36]. Further research including field experiments at Casey has demonstrated causal links between contaminants such as oil and hydrocarbons and differences in benthic communities [34,41,43,94]. Given the multiple contaminants and the magnitude of their concentrations, which in many cases exceed sediment quality guidelines, the likelihood of ecological impacts is high, as has been noted in past studies [6,39], and these are likely to be ongoing.

It is important to understand how the impacts of human activity including pollution will affect coastal regions around stations, in order to differentiate them from other sources of environmental change, and understand potential synergistic interactions. Research on sub-Antarctic marine invertebrates, for instance, has demonstrated some species have increased sensitivity to copper when exposed to increased temperature and/or decrease salinity [95]. Many Antarctic marine invertebrates are known to have narrow physiological tolerance ranges to temperature and salinity [96]. Thus if the physical environment is modified by climate change, organisms may be exposed to stressful environmental conditions, reducing their ability to tolerate elevated pollutant levels. Furthermore, Antarctic marine invertebrates may already have higher levels of sensitivity to contaminants than their counterparts from other regions, due to their slower development rates, which create potential for longer exposures during sensitive larval stages [97].

Contamination of marine sediments around Casey is likely to be confined to the relatively small areas examined in this study in close proximity to the station, consisting of Brown Bay, Shannon Bay, the Casey Wharf and adjacent to the Wilkes disposal site. There may be environmental pollution in areas further afield or between these sites, but this has not been examined. As noted by Kennicutt, Klein [98], however, the spatial extent of contamination and its effects at Antarctic stations is likely to be highly localized and confined to within a few kilometres of the station. Given the relatively calm nature of Antarctic coastal sub-marine environments, due to sea-ice inhibiting waves and wind-driven turbulence and mixing, and generally low current velocities, there is also relatively limited potential for redistribution of contaminants further afield. The main driver of redistribution is likely to be iceberg scour of the sea floor, which varies in its intensity around different regions of the Antarctic coast.

The extent of wildlife interactions with the contaminated sediments or with other fauna in contact with contaminated sediments is unknown. None of the impacted areas at Casey have any regular or resident vertebrate wildlife such as penguins or seals, although they are occasionally observed, but they all have local fish communities. Significant effects on the health of benthic fish species have been observed at Davis Station and hypothesised to be related to wastewater discharges [99]. Effects on benthic communities of marine invertebrates and macroalgae at Casey will be explored in future work.

### Monitoring

The Antarctic Treaty, through the Protocol on Environmental Protection, sets forth the obligations of the Treaty Parties to protect the environment, which are usually enacted through national legislation. The Protocol (Article 3 –Environmental Principles) requires that activities in the Antarctic Treaty area are planned and conducted on the basis of information to allow assessments of their possible impacts on the environment and associated ecosystems. It specifically refers to monitoring and suggests that regular and effective monitoring is conducted to allow assessment of the impacts of ongoing activities, including: the verification of predicted impacts; to provide early warning of any adverse effects of activities; to facilitate early detection of unforeseen effects of activities; and to allow the modification of operating procedures given the results of monitoring or of increased knowledge of the Antarctic environment. Yet published scientific studies describing monitoring and impacts of national Antarctic programmes are very limited, and for most Antarctic stations there are no available data [100]. Monitoring programs such as that conducted by the USA [98] and opportunistic monitoring, as represented in this study, should be undertaken for all Antarctic stations and large infrastructure [100]. The monitoring described in this study was not part of a coordinated long-term monitoring program, but was a result of opportunistic sampling and a series of projects investigating human impacts of specific activities. A range of factors may contribute to the widespread lack of monitoring and reporting by many Treaty Parties including: a lack of monitoring expertise; insufficient funding; and the lack of importance or prestige ascribed to monitoring by funding bodies, particularly compared to research areas rated as higher priority for national Antarctic programs [100]. As stated by [100] “*Until all Antarctic Treaty nations engage with their monitoring obligations and develop together a co-ordinated continent-wide view of human impacts*, *Antarctica’s environmental values will remain under threat of continued degradation and the principles of the Antarctic Treaty brought into disrepute*.”

### Spatial variation and sampling design

The physical and chemical characteristics of Antarctic marine sediments can be highly spatially variable [7,18]. Variation in contaminants is likely to be partly due to the processes responsible for contaminant input in the marine environment. In some instances, contamination may be due to a discrete item on the seafloor (e.g. oil drum or metal object), which would result in elevated levels in its immediate vicinity (i.e. a contamination ‘hotspot’). Physical environmental processes also influence the deposition of contaminated particulate matter, for example, hydrodynamic conditions may result in higher deposition rates in some areas than others. Spatial differences in physicochemical parameters such as redox (Eh), grain size and organic content may also direct the precipitation or adsorption of contaminants into specific areas or depths of the sediment, or affect their measurement. For example, hypoxic patches of sediment are known to occur in Antarctic coastal environments, due to pooling of hypersaline brine over winter in seabed depressions, which leads to reduced Eh and increased availability of metals such as iron within these patches compared to outside them [84]. This process may be responsible for the higher than normal concentrations of cadmium and arsenic that were measured in 2014 at McGrady Cove and O’Brien Bay-3, where such patches have been observed (notable as white and grey patches on seafloor).

Studies investigating potential human induced changes are more powerful, and ultimately more useful, when able to differentiate such effects from natural background variability. As observed in this study, there can be significant spatial variation of grain size and contaminants at small scales (10 and 100 m) within larger areas being compared. The largest scale of comparison, locations, was responsible for the majority of variation, and although smaller scales were generally responsible for less variation, they frequently contributed significant variability that could confound comparisons at larger scales. Estimates of contaminant concentrations within a single site can vary over one to two orders of magnitude. There was also notably high residual variation between replicate samples for most variables measured. This variation demonstrates that monitoring in Antarctic marine sediments requires replication at multiple spatial scales for impact assessment purposes or comparisons with other areas. An effective way to reduce residual variation is by increasing the number of replicates, with larger sample sizes providing more representative data and reducing the impact of random variation, improving the precision of estimates and increasing statistical power. To enable rigorous statistical comparison of contaminant concentrations between different areas, appropriate statistical models are required, such as hierarchical models, or mixed-effects models, which can effectively address residual variation and provide more accurate estimates of variance components. Estimation of variances at appropriate spatial scales can be incorporated in the sampling design, with multiple scales and replicate sampling at each scale [101,102]. Replication effort may be constrained by the costs of analysis, but as analytical technologies have improved their costs have decreased, increasing the degree of replication possible for the same cost, particularly for expensive analyses such as for PCBs and PBDEs. Logistical constraints on collecting samples in Antarctica can also be prohibitive, and the opportunity to do field work can be sporadic and subject to unforeseen changes.

### Influence of analytical methods on measurement of contamination

Consideration of analytical methods is vital when designing a contamination study and efforts to standardise methods across studies to achieve consistency of data is encouraged. For example, the method used to digest sediment and extract metals for analysis can have a major influence on the comparison of metal concentrations among different studies. A partial extraction, employing digestion with dilute acid (e.g. 1 M HCl) gives an estimate of the bioavailable fraction, whereas a total extraction with concentrated acid dissolves all (or most) of the metals from the mineral matrix, including those not normally available to biota and therefore unlikely to pose an environmental risk. This can confound assessments of anthropogenic contamination in marine sediments [19]. In partial extractions, extraction time will also influence the measurement of metal concentrations [46], as seen for a number of metals in this study, including manganese, iron, chromium and nickel. In this investigation with 1 M HCl, an extraction time of 4 h was mostly used, but 30 min and 1 h were employed for some early analyses. While this likely had an effect on the magnitude of some of the measured concentrations (and probably more so for control samples), we have concluded it did not have a major influence on the interpretation of chemical patterns observed in this study, especially given the high level of heterogeneity observed at the different spatial scales.

Many investigations utilise strong acid digestion methods, and while this approach can be precisely controlled and data of high reproducibility is obtained, results mix anthropogenic inputs or bioavailable fractions with the strongly bound mineral fractions. This can be compensated for to distinguish anthropogenic contamination from geogenic or background composition by using normalization methods based on grain size fraction or conservative elements such as aluminium or lithium. In contrast, an operationally defined partial extraction method, even with its greater dependence on experimental parameters, can more readily provide this differentiation. From a practical perspective, these techniques offer an advantage as they are often simpler, quicker and less hazardous to perform. With all these considerations, we recommend a 4 hour extraction using 1 M HCl on the <2 mm sediment fraction [19,46].

### Mitigation and remediation

Preventing contamination of the marine environment needs to be more thoroughly considered at Antarctic stations. Some of the sources of contamination are relatively easy to mitigate: for example, modern wastewater treatment technologies can produce ultra clean water that poses no environmental risk. However, current station wastewater treatment practices generally lag well behind technological advances [103], in addition to being inferior to domestic treatment practices in many cases. Improvements to environmental management systems would also help to limit contamination from station sources, such as those posed by fuel storage and transfer. Hydrocarbon spills and leaks are relatively common at most stations. Between 1988 and 1999, 93 hydrocarbon spills greater than the 200 L reporting limit were reported to the Council of Managers of National Antarctic Programs (COMNAP), with a further 58 incidents between 1999 and 2000 [104,105]. At a single Australian station 38 spills of diesel fuel were reported between 2008 and 2018 (total volume of >14632 L) [106]. At McMurdo station 385 spills (mostly aviation kerosene) were reported between 1991 and 2000, with one spill of 260000 L [98,107]. The Australian Antarctic Program has implemented extensive remediation to clean up spills at Australian Stations. Some contamination sources, however, are due to legacy practices, such as the dumping of waste into landfill sites, which is no longer allowed under the Treaty. There exist many legacy contaminated sites around Antarctica [22] that pose varying degrees of environmental risk, and dealing with these sites is a more complex issue [14].

Annex VI of the Environmental Protocol, which is yet to be ratified by the Consultative Parties, deals with liability arising from environmental emergencies related to scientific research programs, tourism and all activities in the Antarctic Treaty. Antarctic operators will be required to undertake preventative measures and to establish contingency plans for activities with potential adverse impacts on the Antarctic environment. When environmental emergencies occur, operators will be required to take prompt and effective response action, including liability for the costs.

### Conclusions

A legacy of environmental pollution exists in marine habitats around Casey Station. Despite improved environmental management practices over the past 20–30 years, contaminants in marine sediments in disturbed locations have remained at similar levels or are increasing. Marine sediments are known to be sinks for pollutants entering the ocean, particularly those with high proportions of fine grains where contaminants can be bound. Thus, even small and infrequent inputs could lead to localised increases in sediment contaminants. There is also some evidence that contaminated sediments may be redistributed as a consequence of sediment resuspension and transport, extending the spatial extent of contamination.

Casey Station could be considered fairly typical of many established Antarctic research stations, particularly of the 44 established prior to 1980, and also many of the 51 established since. There are 62 research stations situated in coastal areas [2], some of which are highly likely to have similar contamination profiles in local marine environments, greatly expanding the estimated footprint of human presence in Antarctica, and the extent of environmental impacts on marine ecosystems. Several types of contaminants are recommended as potential markers of contamination of marine environments around stations including metals (lead, copper, zinc, tin, cadmium), hydrocarbons (TPH) and PBDEs. This study provides evidence to support greater continent wide monitoring efforts, and to raise the awareness of the potential impacts of research stations on the Antarctic environment, and inform environmental management practices.

## Supporting information

S1 Table Summary of analytical details and QC parameters for the 1 M HCl data sets. (DOCX)

S1 File Chemical analysis methods. (DOCX)

S2 File Effect of acid extraction time. (DOCX)
