# Supplementary material for: Contamination of the marine environment by Antarctic research stations: Monitoring marine pollution at Casey station from 1997 to 2015
Source: PLoS One. 2023 Aug 9;18(8):e0288485. doi: 10.1371/journal.pone.0288485 (PMC10411823; doi:10.1371/journal.pone.0288485)
Supplement: S1 Table — (DOCX) [file pone.0288485.s001.docx]

#### Supplementary Information - Contamination of the marine environment by Antarctic research stations: monitoring marine pollution at Casey station from 1997 to 2015

#### Table S1. Summary of analytical details and QC parameters for the 1 M HCl data sets

| **Analysis set^(1)^** | **Sediment sampling method and preparation** | **Analysis sub-sample** | **Extraction procedure^(2)^**  **and time** | **Analytical technique** | **Data correction** | **LOQ^(3)^** | **Precision for CRMs^(4)^** | **Precision for samples^(4)^** |
| --- | --- | --- | --- | --- | --- | --- | --- | --- |
| 1997/98 | Cores;  <2 mm fraction, wet sieved, dried at 103 °C | Composite of 0-5 cm section (homogenised),  5 g dry sediment | A / 0.5 h and 4 h | Quadrupole ICP-MS (AGAL) | No blank-correction (procedural blanks generally low level) | Extract: 1-5 µg L^-1^  Dry sediment:  20-100 µg kg^-1^ | Duplicates in sets 1 and 2: MESS-2: 2±1 & 2±5 %; PACS-2: 9±3, 5±4 %; n=11, 12 metals, resp. | Set 1: Two sets of Duplicates, 10±3 & 18±9 %, n=10 metals  Set 2: single sample n=5 reps, 18±11%, n=10 metals |
| 1998/99 (BBG) | Cores;  <2 mm fraction, wet sieved, dried at 103 °C | Composite of 0-5 cm section (homogenised),  5 g dry sediment | A / 1 h | Quadrupole ICP-MS (AGAL) | Only Sb & Cr blank-corrected (procedural blanks generally low level) | Extract: 1 µg L^-1^  Dry sediment: 20 µg kg^-1^ | Pair of Duplicates: MESS-2: 5±6 % (mostly ≤7%), PACS-2: 1.4±1.4 % (mostly <1%); n=11, 12 metals, resp. | Seven sets of Duplicates: 6±2 % (range 4-10 %), n=12 metals |
| 2005/06, 2006/07 (analysis 2008) | Cores (upper part sectioned in 1 cm intervals);  <2 mm fraction, wet sieved, dried at 60 °C | Composite of 0-5 cm section (homogenised) from 1 cm sections,  2 g dry sediment | B / 4 h | High resolution ICP-MS (CSL) | Extract data blank-corrected w/ procedural blank values | Extract: ≤1 µg L^-1^ (most metals), several 4-40 µg L^-1^  Dry sediment: 20 µg kg^-1^ (most metals), 0.08-0.8 mg kg^‑1^ (others) | Replicates (n=6) of both MESS-3 and PACS-2:  both 5±3 %, n=28, 30 metals, resp. | No Duplicates (extrn + analysis) but duplicate measurement of 30 extracts (in two separate ICP-MS runs): 2.4±1.1 % (range 1-6 %), n=30 metals |
| 2014/15 | Cores (0-5 cm section);  whole sediment (coarse material >2 mm selectively removed), subsampled at time of sectioning | Composite of 0-5 cm section (homogenised),  ~3 g wet sediment (equivalent to 0.3-2 g dry wt) | B / 4 h | ICP-AES (AAD)  (High resolution ICP-MS (CSL) for verificat-ion subset: agreement ±10% most metals) | Extract data not blank-corrected; Ag calc from post-run calibn, Sb MDL recalc, Cd corrected for As interference | Extract: 2-20 µg L^-1^ (most metals), several incl Fe 20-40 µg L^-1^  Dry sediment:  0.07-0.7 mg kg^-1^ (most metals), 0.7-1.3 mg kg^-1^ (others) | Pair of Duplicates: MESS-3: 3±4 %, PACS-2: 0.8±0.8 %, n=25, 24 metals, resp. | Six sets of Duplicates: 4±2% (range 1-7%), n=22 metals  This was ~5x less precise than ICP-AES measurement alone i.e. duplicate measurement of single extract: 0.7±0.6 % (range 0.2-3%) for 10 Duplicates, 22 metals |

(1) Data for a small number of sediment grab samples collected in 1996/97 were also included in this investigation. Analytical method information is sparse but subsamples of size-fractionated (<2 mm?) sediment were extracted with 1 M *nitric acid* for 0.5 h using a procedure similar to (A) below and analysis by ICP-MS (PerkinElmer Elan 6000) at Macquarie University, NSW (LOQ 1-10 µg L^-1^). (2) Extraction procedure: A) Sample mass ±0.1 mg. Extraction in acid-washed/Milli-Q water-rinsed 250 ml Teflon bottles on platform shaker, 1:20 w/v extraction ratio (dry sediment), extractant volume ±0.5 ml. Filtration using plastic vacuum filter system with 0.45 µm cellulose acetate or cellulose nitrate membrane filters (Sartorius); B) Sample mass ±0.1 mg. Extraction in new (but not pre-cleaned) 50 ml polypropylene Sarstedt vials on rotary mixer (~50 rpm), 1:20 (dry) or 1:10 (wet) w/w extraction ratio (wet sediment ~0.5 DMF), extractant volume weighed (±0.1 mg); centrifugation before filtration using 0.45 µm cellulose acetate cartridge filter (Sartorius) fitted to disposable plastic syringe. More precise control of extraction time and filtration time. (3) Limit of Quantitation (LOQ) is the reported value or range from laboratory or calculated as LOQ = 2.5 x MDL_99%_ (method detection limit at 99% confidence level); values for sediment derived using mean dry sample and mean extract weights. (4) Precision data for replicate (mostly duplicate) extraction and analysis of CRMs and samples: relative standard deviation (RSD) values except for 1998/99 (% average deviation); table contains mean value (± std dev) and range (for samples only).
