# Supplementary material for: Contamination of the marine environment by Antarctic research stations: Monitoring marine pollution at Casey station from 1997 to 2015
Source: PLoS One. 2023 Aug 9;18(8):e0288485. doi: 10.1371/journal.pone.0288485 (PMC10411823; doi:10.1371/journal.pone.0288485)
Supplement: S1 File — (DOCX) [file pone.0288485.s002.docx]

# Supporting Information S1 - Chemical analysis methods

# Contamination of the marine environment by Antarctic research stations: monitoring marine pollution at Casey station from 1997 to 2015

**Jonathan S Stark*^1^, Glenn J Johnstone^1^, Catherine King^1^, Tania Raymond^1^, Allison Rutter^2^, Scott C Stark, Ashley T Townsend^3^**

1. Environmental Protection Program, Australian Antarctic Division, Australia
2. Analytical Services Unit, Queens University, Canada
3. Central Science Laboratory, University of Tasmania, Australia

# Chemical analysis methods

## Water-extractable nutrients in sediment by flow injection analysis (FIA)

Wet sediment (5 g) subsampled from the roughly homogenised, unsieved 0-1 cm core section, was extracted with 25 ml of Milli-Q deionised water (1:5 w/v equivalent to ~1:10 w/v extraction of dry sediment) in 50 ml polypropylene centrifuge tubes (Sarstedt) at room temperature (~20 °C) for 1 h on a Ratek rotary mixer set at ~50 rpm. At completion, mixtures were centrifuged at 1500 rpm for 20 min prior to filtration through a Sartorius cartridge filter: without pre-filter (Minisart NML) for half of the samples (2 or 3 cartridges required per sample), with a glass fibre prefilter (Minisart NML Plus) for the remainder. Samples were frozen for preservation until analysis.

Analysis of ammonia, NO_x_ (nitrite and nitrate) and dissolved reactive phosphate in extracts was performed at Analytical Services Tasmania (AST, New Town, TAS) using a Lachat QC8500 flow injection analyser (FIA) running AST method 1205. This is based on American Public Health Association (APHA) standard colorimetric methods (2005) 4500-NH_3_ H (indophenol blue), 4500-NO_3_^-^ I (Cd reduction to nitrite with formation of red-purple azo dye) and 4500-P G (molybdo blue dye). Extracts were diluted 1:5 v/v with deionised water to minimise interference from chloride in the Cd reduction step for nitrate. Laboratory method reporting limits (MRLs) were 25 (NH_3_), 10 (NO_x_) and 15 (PO_4_^3-^) µg N or P L^-1^; these were converted to mg/kg data on a dry weight basis after correcting the extractant volume (by weight) for the water content of the sample. Duplicate samples and QC standards were measured at 5% frequency with an acceptable precision limit of ±10% while sample recovery tests were acceptable at 100 ± 20%.

Data obtained for a nutrient certified reference material (CRM) (NCS DC 85101a Available Nutrients in Soil, China National Analysis Centre for Iron and Steel, 2009) extracted/analysed along with the samples were consistent with the long-term data accumulated for this standard in our laboratory. The analytical precision for the four nutrient species in the CRM (n=4) ranged from 5-12% (relative standard deviation, RSD). This was superior to that measured for n=8 sample duplicates (extraction/analysis) which averaged 10-17% RSD, reflecting the greater heterogeneity of the samples. For additional details refer to [doi:10.26179/5df6d4a4958bb](http://dx.doi.org/doi:10.26179/5df6d4a4958bb).

## Total organic matter in sediment by loss on ignition (LOI)

The LOI analysis was performed on a subsample of homogenised wet sediment sufficient to yield at least 1‑2 g dry material, estimated from dry matter fraction (DMF) data determined from the 1 M HCl extraction residues: on average this was 5 ± 2 g (range 1.4-10 g) wet sediment. Samples in small, clean and dry porcelain crucibles were weighed (±0.1 mg), covered with lids and placed into a drying oven (Contherm Thermotec 2150). The temperature was ramped from room temperature at 0.5 °C min^-1^ to 105 °C to allow most of the water to evaporate from samples without spluttering and potential loss of the wet sediment (low DMF samples were slurries). Following drying overnight (typically ~18 h), crucibles were cooled in a low humidity cabinet and weighed to calculate the DMF. Measurement of n=10 duplicates was at an average precision of ±1.5% (RSD) which was inferior to that found for n=4 duplicates of the more homogeneous and high DMF (97-98%) CRMs: MESS-3 (±0.14%) and PACS-2 (±0.32%).

Crucibles (with lids) were then loaded onto a stainless steel tray and placed into an electric muffle furnace (Modutemp WE342BM01) and ignited at 550 °C for 4 h. After cooling to ~100 °C the crucibles were moved to the low humidity cabinet to reach room temperature and reweighed (without lids) to calculate LOI. Precision of duplicate samples averaged 4% (RSD) (n=10, range 1.0-14%) which was consistent with that measured for n=4 duplicate pairs of the CRMs (7 and 11% for MESS-3 and PACS-2, respectively). For additional details refer to [doi:10.26179/5df6d4a4958bb](http://dx.doi.org/doi:10.26179/5df6d4a4958bb).

## Total petroleum hydrocarbons (TPH) and persistent organic pollutants (POPs) in sediment

Frozen sediment samples for the determination of TPH and persistent organic pollutants (POPs) were transported by air freight from Tasmania to the Analytical Services Unit (ASU), Queen’s University, Kingston, Ontario, Canada and kept at low temperature until the time of analysis.

### TPH

Analysis of TPH in samples from the 1998/99 Brown Bay Grid study is described in Stark et al. (2005). For other samples, analysis of TPH fractions F1 (C_6_-C_10_), F2 (C_10_-C_16_), F3 (C_16_-C_34_) and F4 (C_34_-C_50_) was performed as prescribed in the Canadian Council of Ministers of the Environment) (CCME) Reference Method for Canada Wide Standard for Petroleum Hydrocarbons in Soil, June 2000.

### Fraction F1 (C_6_-C_10_)

Accurately weighed samples of wet sediment (5 g) were extracted with 10 ml of methanol on a shaker for 1 h. Methanol extracts were transferred into clean vials and refrigerated until analysis. A 0.4 mL aliquot of each extract was diluted to 2 ml with deionised water and analysed by SPME (solid phase microextraction) GC/FID (gas chromatography with flame ionization detection). Retention time marking was done using nC_6_ and nC_10_ hydrocarbons and calibration with toluene. Blanks, control samples and duplicates were run at a frequency of approximately 20%. Sample DMF was determined for a subsample of sediment and the final concentration result (µg g^-1^) calculated on a dry matter basis (DMB).

### Fractions F2, F3, and F4 (C_10_-C_50_)

Soil samples were homogenized and subsamples dried for moisture determination. Accurately weighed samples of wet sediment (10 g) were extracted using 50 ml of 1:1 v/v hexane/acetone (3x 50 ml) with sonication. The combined extracts were filtered through anhydrous Na_2_SO_4_ and 3 ml of toluene added. The extract was then concentrated by rotary evaporation and transferred to a 50 ml tube. Following dilution to 30 ml with 1:1 v/v hexane/dichloromethane, silica was added and the sample shaken for 5 min. Once the mixture had settled, a subsample of the extract was placed into a GC vial and analysed by GC/FID. Blanks, control samples and duplicates were run at a frequency of approximately 20%. Calibration and retention time marking was done using nC_10_, nC_16_, nC_34_ and C_50_ hydrocarbons and the final concentration result (µg g^-1^ DMB) reported for each fraction.

Analytical precision was assessed by replicate measurement of a lab control standard and samples. For the control standard (n=2, 4) this was better than 10% RSD for both the F1 and F2-F4 (combined) fractions and analyte recovery averaged 90-100%. For the samples, precision was poorer. Where it could be quantified for F3 and F4 fractions (all F1 and most F2 data <RL), duplicate extraction and analysis of samples in the laboratory had an average precision of 30% RSD (range 10-70%). For duplicate 2014/15 samples, however (i.e. sediment subsampled twice at AAD for TPH during processing of the 0-5 cm core section), precision was 50-120% RSD. This is based on more limited data, but likely reflects a greater heterogeneity imparted to subsamples by this process at the AAD compared to duplicate subsampling carried out at ASU.

## Polybrominated diphenyl ethers (PBDEs) and total polychlorinated biphenyls (PCBs) in sediments

Samples were air-dried overnight at room temperature and spiked with the surrogate decachlororbiphenyl. Sodium sulphate and Ottawa sand were added to each accurately weighed sediment sample (1-5 g dry weight). Samples were extracted with dichloromethane (DCM) using the Soxhlet method and extracts concentrated by rotary evaporation. Because the sediments contained a large amount of plant material (seaweed), extracts were applied to a gel permeation chromatography column (70 g S-X3 BioBead stationary phase, DCM mobile phase) for cleanup. The extract fraction containing PBDEs and PCBs was solvent exchanged with hexanes, applied to a Florisil cleanup column and concentrated down to a final volume of 1.0 ml.

Total PCBs (ng g^-1^ DMB) were analysed by GC-ECD (gas chromatography with electron capture detection) using an Agilent 7890A instrument and a Supelco SPB-1 column (30 m, 0.25 mm i.d. X 0.25 μm film thickness). Quality control involved replicate analysis (n=3, 4) of a control standard (precision 20% RSD) and duplicate analysis of six samples (12, 15, 74% RSD for the 3 samples > reporting level, RL). Recovery of standard additions to the control was acceptable (100, 111%).

PBDEs (ng g^-1^ DMB) were analysed by GC/MS/MS (gas chromatography with tandem mass spectroscopy) using a Varian 4000 GC-MS and an Agilent DB-1 MS capillary column (30 m, 0.32 mm i.d. x 0.1 μm film thickness). Varian MS Workstation V.6 software was employed to quantify 26 of the 209 PDBE congeners (PBDE-3, 7, 15, 17, 28, 47, 49/71, 66, 77, 85, 99, 100, 119, 126, 138, 153, 154, 156, 183, 184, 191, 196, 197, 206, 207, 209). Replicate analysis (n=5) of 9 congeners in a control standard was at a precision of 4-11% (RSD) and analyte recovery 100-127% (5 congeners >RL). For five sample duplicates, analytical precision for the same 9 congeners (where >RL) averaged 20-40% RSD (range 2-60%).

## Elements in sediment – partial digests with 1 M hydrochloric acid (HCl)

The 1 M HCl elemental (‘metals’) data constitute the most extensive data set examined in this study but they are also subject to the greatest variation in experimental parameters. Some of this variance will have little to no effect on the data produced and its interpretation whereas other components may have significant influence. This will be discussed further following description of the analytical procedures, which are summarised for the different data sets along with quality control (QC) information in Table S1.

A 2-5 g subsample of wet or dry sediment was extracted with an appropriate volume of 1 M HCl to achieve a 1:10 or 1:20 w/v extraction for wet and dry sediment, respectively (1:10 w/v for wet sediment based on average DMF of 50%). Concentrated HCl acid (32-36% w/w) of analytical grade quality or higher (e.g. BDH Aristar) was diluted with Milli-Q deionised water (~1:10 v/v) to prepare the 1 M HCl extractant.

For the earlier data sets, 1996-1999 (extraction procedure A): Sediment samples were wet-sieved through 2 mm nylon mesh and oven-dried at 103 °C. A 5 g subsample of dry sediment was mixed with 100 ml of extractant in a clean 250 ml Teflon bottle and agitated on a platform shaker for 0.5 or 1 h. At completion of extraction the mixtures were filtered through 0.45 µm cellulose acetate or cellulose nitrate filters (47 mm diameter, Sartorius) using a Sartorius polycarbonate vacuum filtration unit. Extracts were transferred to clean plastic tubes and refrigerated until analysis by Inductively Coupled Plasma Mass Spectrometry (ICP-MS).

For the later data sets, 2005 onward (extraction procedure B): Sediment samples were wet sieved at 2 mm and dried as above or (2014/15) the 0-5 cm sediment core sections were simply homogenised and subsampled (with larger pebbles and shell material excluded). Typically 2 g of dry or 3 g of wet sediment were combined with 40 or 30 ml (weighed) of extractant solution, respectively, in a 50 ml polypropylene centrifuge tube (Sarstedt) and mixed at room temperature (~20 °C) for 4 h on a Ratek rotary tube mixer set at ~50 rpm. The mixtures were centrifuged at 1500 rpm for 20 min and then filtered through a Sartorius cartridge filter (Minisart NML, usually 2 filters required per sample) into a clean tube. Extracts were refrigerated until analysis by ICP-MS or ICP-AES (Atomic Emission Spectroscopy).

Concentration data for sediments were calculated on a dry matter basis (mg kg^-1^ DMB) from the ICP data for the extracts (µg L^-1^). For wet sediment samples, extractant volume (by weight) was corrected for the water content of the sample and DMF values were determined by drying the extraction residues or separate subsamples. Concentration data were not corrected for the elemental contributions from sea salt in the marine sediment but this is only significant for elements that are major components of seawater (i.e. Na, Mg, Ca, K, Sr and S, where measured).

Each discrete analysis set, during the extraction and analysis stages, was subject to the QC procedures employed by the specific laboratories involved to quantify and minimise analytical uncertainty. In addition, to provide a measure of QC across these data sets generated by different labs/instruments/operators over a considerable time period, we employed two marine sediment CRMs sourced from the National Research Council Canada (NRCC): MESS-2/MESS-3 (MESS-2 used initially (1996-99) then replaced by the near-identical MESS-3) and PACS-2. MESS-2/3 and PACS-2 are representative of a pristine and moderately contaminated marine sediment, respectively. Replicate extraction and analysis of the CRMs was performed to generate at least duplicate data for each CRM in every analysis set, and the results assessed for accuracy and precision. For the later data sets in particular, this QC process was facilitated by comparison with reference data compiled by our analytical program in collaboration with the main ICP-MS facility employed (Townsend et al. 2007).

### Quality control of ICP-MS and AES analyses

Quality control information for the ICP-MS analyses of the earlier data sets (1996-99) is relatively sparse compared to that available for later analyses because the former were done on a strictly commercial basis while the latter were undertaken collaboratively with the service provider or in-house by the authors. However, we have a high level of confidence in the quality assurance program of the NATA-accredited laboratory involved and therefore with the quality of the data obtained.

Method references for the data sets:

- Data 1996-1999 are from analyses performed at the Australian Government Analytical Laboratories (AGAL), Pymble, NSW using a PerkinElmer Elan 6100-DRC Quadrupole ICP-MS. Analytical details (specifically for the 1998/99 Brown Bay Grid data) are described in Stark et al. (2005).
- Data 2005-2007 are from analyses performed at the Central Science Laboratory (CSL), University of Tasmania (UTAS), Sandy Bay, TAS using a Thermo Finnigan ELEMENT 1 Sector Field (or High Resolution) ICP-MS, as described in Townsend (2000) and Townsend et al. (2007).
- Data for 2014/15 were measured in the Wild Laboratory, Australian Antarctic Division (AAD), Kingston, TAS using a Varian 720-ES ICP-AES as described in Fryirs et al. et al (2015). A verification subset of extracts was analysed at CSL-UTAS with a Thermo Fisher ELEMENT 2 Sector Field ICP-MS.

For the ICP-MS analyses at CSL-UTAS, extracts were diluted 1:10 v/v with Milli-Q deionised water and spiked with indium (In) internal standard (100 µg L^-1^) prior to analysis. QC procedures included regular measurement of rinse blanks, a 10 or 100 µg L^-1^ calibration check standard and the NIST (National Institute of Standards and Technology, USA) 1640 or 1640a (‘Trace elements in natural water’) standard reference material (SRM). Recovery tests, typically involving multi-element standard addition at 100 µg L^-1^ were also performed on extracts. Data for procedural blanks prepared during the extractions were used to correct measurements of samples.

For the ICP-AES analyses, neat samples were mixed 1:1 v/v in-line with a CsCl matrix modifier (0.75% w/v in 10% v/v HNO_3_) spiked with Y internal standard (5 mg L^-1^) prior to the nebulisation step. QC measurements included rinse blanks, calibration check standards (0.1 and 10 mg L^-1^), and a low concentration water CRM (QCP-MTL, Inorganic Ventures, USA). Duplicate measurements of extracts and QC standards were performed but not recovery tests. Procedural blanks were measured but data were not blank-subtracted. Method detection limits (MDLs) were calculated from n=8 replicates of a 20 µg/L standard.

Quality control data for the ICP-MS and -AES analyses are summarised in Table S1.

**Estimation and correction of Ag, Sb, Cd in the 2014/15 ICP-AES data set**

Silver was absent from the calibration standard employed but its optical emission intensity was measured at two wavelengths. ICP-AES calibration of Ag was achieved post-analysis using Ag concentration data for the water CRM (QCP-MTL) and that measured by ICP-MS for the verification subset (6 samples) and the marine sediment CRMs. The more sensitive line (328.1 nm) was used to quantify Ag in the ICP-AES data set. However, in addition to analytical limitations owing to the relatively poor stability of Ag in the HCl digest, anomalous values of emission intensity or poor reproducibility were measured for some of the calibration extracts, and so the Ag data should be considered with low confidence as semi-quantitative only.

Like in the other data sets, Sb was measured at levels close to the limit of quantitation (LOQ) or reporting limit (RL). Initial calculation of the RL based on mean sample weight and extraction volume (as for the other elements/analysis sets) resulted in the majority of Sb data <RL. To improve statistical comparison with data from ICP-MS analyses with lower RLs, the ICP-AES RL for Sb was recalculated using actual sample weight to a (typically lower) sample-specific value. This enabled the majority of Sb data to be reported at a value >RL (and not at 0.5 x RL). Further details are found at [doi:10.26179/5df6d4a4958bb](http://dx.doi.org/doi:10.26179/5df6d4a4958bb).

Cadmium was subject to positive bias (overestimated) in the ICP-AES analysis owing to spectral interference from As (Lambkin and Alloway 2000; McBride 2011). This was evident in comparison with the ICP-MS data for the verification subset of samples. Consequently, Cd concentration values were corrected using a linear correlation between the difference in the ICP-MS and -AES data and the As concentration. Details are summarised at [doi:10.26179/5df6d4a4958bb](http://dx.doi.org/doi:10.26179/5df6d4a4958bb).
