# Supplementary material for: Contamination of the marine environment by Antarctic research stations: Monitoring marine pollution at Casey station from 1997 to 2015
Source: PLoS One. 2023 Aug 9;18(8):e0288485. doi: 10.1371/journal.pone.0288485 (PMC10411823; doi:10.1371/journal.pone.0288485)
Supplement: S2 File — (DOCX) [file pone.0288485.s003.docx]

# Supporting Information S2 – Effect of acid extraction time

# Contamination of the marine environment by Antarctic research stations: monitoring marine pollution at Casey station from 1997 to 2015

**Jonathan S Stark*^1^, Glenn J Johnstone^1^, Catherine King^1^, Tania Raymond^1^, Allison Rutter^2^, Scott C Stark, Ashley T Townsend^3^**

1. Environmental Protection Program, Australian Antarctic Division, Australia
2. Analytical Services Unit, Queens University, Canada
3. Central Science Laboratory, University of Tasmania, Australia

### Evaluating the effect of acid extraction time on measurement of elements in sediment

#### Comparison with Certified Reference Materials (CRM)

Factors of the dilute HCl acid partial extraction of sediment affecting the proportion of an element extracted from the sediment and its reproducibility include: the concentration of extractant, the ratio of sample mass to extractant volume, temperature and time; i.e. parameters influencing the position of equilibrium or the kinetics of the various reactions. Of these, extraction time is the factor subject to most variation across the data sets: 4 h was mostly employed (2005-07, 14/15) but 0.5 h (1996-98) and 1 h (1998/99) were used to generate the earlier data. Other sources of variation include extraction of dry or wet sediment samples and the differences in mass to volume ratios associated with this (1/20 vs ~1/10 w/v, respectively), and small differences (±5-10%) in the concentration of the 1 M HCl extractant. Extractions were all carried out at room temperature (typically 20 ± 1-2 °C) with mixing achieved (for earlier data) by platform shaking or (more vigorously for 2005 onward) with a rotary tumbler.

Differences in analytical measurement technique based on instrument type, laboratory or analyst may also introduce systematic bias or random uncertainty (‘noise’) into a data set. For these data sets, collected over two decades at different labs with different instruments (predominantly ICP-MS) this should be considered. In the 2014/15 ICP-AES analysis, for instance, bias in the Cd data was identified on comparison with ICP-MS verification data and subsequently corrected (as described above). Generally, however, we can be confident that all analyses were performed under conditions controlled by robust quality assurance programs ensuring data of high and defendable accuracy.

The influence of time on the extraction of metals from sediment by 1 M HCl was investigated by Snape et al. (2004). In this study, uncontaminated (pristine) sediments (MESS-2 and a sample from O’Brien Bay, Casey) and contaminated sediments (PACS-2 and a sample from Brown Bay, Casey) were extracted over different periods ranging from 15 min to 24 h. Extraction efficiency as a function of time varied with elements but kinetic behaviour, overall, was controlled by whether the source of an element was primarily natural (geogenic) or anthropogenic. In contaminated sediments, anthropogenic metals will be located predominantly in the more labile phases associated with faster kinetics of extraction. In contrast, for pristine sediments, many elements are present mainly in the more resistant phases of the sediment matrix and are leached relatively slowly over time (equilibrium may not be reached even after 24 h of digestion). Although this is a simplified view of selective extraction and sediment composition (e.g. geogenic and anthropogenic metals can be located in phases with moderate extraction kinetics; kinetics can be complicated by processes such as re-adsorption, observed for Sb and As), this model can be used to explain many of the observed differences in the 1 M HCl extraction of pristine and contaminated sediment.

Many applications of the dilute HCl extraction of sediments have employed digestion or extraction periods of 0.5 -1 h (e.g. Malo 1977, Sutherland 2002) and 1 h is generally recommended in the assessment of contaminated sediment (Simpson and Batley 2016). This was the case for the earlier data sets (1996-99) of our investigation. Short extraction times favour the liberation of metals with faster kinetics (i.e. typically anthropogenic metals from labile phases) and can provide a satisfactory differentiation between pristine and contaminated sediments. However, given the continuum in the kinetic parameters of extraction (very fast to very slow) and also owing to practical limitations in the laboratory (e.g. it can be difficult to constrain precisely the time for which sediment is in contact with extractant when processing extracts of many samples), shorter extraction periods may also be associated with less than maximum extraction efficiency and poorer reproducibility. In Snape et al. (2004) an extraction time of 4 h was shown to be optimal and practical for achieving a near-equilibrium, reproducible recovery of the metals of interest in the Casey marine sediments and CRMs tested, enabling better differentiation between samples from contaminated and uncontaminated control locations. This extraction period was used for all our subsequent measurements of 1 M HCl-extractable metals in marine sediments.

To quantify potential bias from variation in extraction time in the data for the Casey sediments in this investigation and its influence on evaluating changes in contaminant concentrations over time, measurements for 12 elements (Sb, As, Cd, Cr, Cu, Fe, Mn, Pb, Ni, Ag, Sn, Zn) in the MESS-2/3 and PACS-2 CRMs from each data set (Figure S1) were compared along with the reference data of Townsend et al. (2007). The latter were derived from 4 h extractions of the CRMs performed in our research group separate from those accompanying the Casey sediment data sets. Also included are CRM data (4 h, FOCE 2015) generated under similar conditions to the 2014/15 data set (same year/lab) but measured by ICP-MS instead of ICP-AES.

Considering all of the CRM data in Figure S1, the critical observation is that concentrations of elements measured with the shorter extraction times are often significantly less than those for 4 h. For Sb, As, Ag and Sn, there is relatively large variation in data obtained across the whole range of extraction time. This is probably a consequence of the greater analytical uncertainties associated with the determination of these elements, especially in MESS-2/3 (all in the vicinity of the reporting limits but the trend found in Snape et al. (2004) for the metalloids Sb and As to decrease slightly (re-adsorb) over longer periods was also apparent). A positive correlation in extract concentration with time, however, is generally evident at the higher levels measured in PACS-2.

For the other elements (all metals mostly extracted at >1 mg kg^-1^) there is generally a positive correlation between extract concentration and extraction time for both CRMs, but there are subtle differences depending on the element and CRM. Cr and Ni are measured at approximately the same concentration in each CRM and there is a well-defined correlation with time. For Mn, Cd and Cu, the effect of time appears to be greater for PACS than MESS (Cd and Cu extracted at up to ~10x higher concentration with greater efficiency from PACS compared to MESS, but vice-versa for Mn). In contrast, Fe extraction appears to be more time-sensitive for MESS compared to PACS despite similar concentrations and efficiencies. Finally, extraction of Zn and especially Pb (6-10x higher concentration and greater efficiency for PACS compared to MESS) is only weakly time-dependent for both CRMs.

Consistent with the findings of Snape et al. (2004), it is apparent that the effect of time on the dilute HCl extraction of the MESS and PACS sediments is complex and element-dependent, attributable to similarities/differences in element speciation and physical constraints on reaction progress. Some metals, like Pb and Zn, reach equilibrium quickly for ‘uncontaminated’ MESS and ‘contaminated’ PACS, independent of extraction efficiency, suggesting a presence in mainly labile phases in both CRMs. Fe and Ni are extracted at similar concentrations and efficiencies from both CRMs but only Fe from PACS appears time independent. The slower leach of Fe from MESS is likely a reflection of its location predominantly within relatively inert phases of the sediment matrix compared to PACS, whereas it could be inferred that Ni is speciated similarly in both CRMs. In cases where time dependency is more apparent for metals extracted at higher concentration and efficiency from ‘contaminated’ PACS (e.g. Cd and Cu), this may simply be a consequence of physical constraints on the reaction; i.e. same speciation (and kinetic parameters) in the CRMs but it takes longer to extract a larger amount of metal from the same mass of sediment.

Figure S1. Comparison of elemental concentrations determined for the MESS-2/3 (M) and PACS-2 (P) CRMs in the different 1 M HCl partial extraction analysis sets with extraction time varying from 0.5 to 4 h. Concentration data are mg kg^-1^ dry sediment (left-hand axis, log_10_ scale); error bars are standard deviations for the experimental data and the 95% confidence interval for the reference data. Extraction efficiency (proportion of total element concentration extracted, from Townsend et al. 2007) is also shown (right-hand axis). No reference data available for Ag.

If MESS-2/3 and PACS-2 are considered as models of pristine (control) and contaminated (impacted) sediment, respectively, we can conclude:

- Data measured with an extraction time of only 0.5 h may be of lower magnitude and poorer reproducibility than data obtained from longer periods, regardless of whether sediment is pristine or contaminated. This is highlighted by the large difference between 0.5 h and 1 h values (for some elements, the largest difference is between the two 0.5 h data sets, probably because extraction time was controlled imprecisely). Consequently, pre-cleanup data from 1996-98 defined by 0.5 h extraction likely underestimates background concentrations in control samples or contaminant levels in impacted sediments compared to later data generated by longer extraction. The CRM data indicates that 0.5 h values may be 20-60% lower than 4 h data, with the effect slightly less for pristine compared to contaminated sediment (median bias for PACS and MESS -50% and -30%, respectively). This would compound error and imprecision in the calculation of concentration ratios or enrichment factors. If the PACS/MESS elemental ratios are used to model the concentration ratios (CR) of a contaminated sediment relative to its background control, the error (% deviation) in the 0.5 h CR is estimated to be approximately +30 to -60% relative to the 4 h CR (reference data) for most of the elements of interest. This is illustrated in Figure S2.
- Data obtained for 1 h extraction of the CRMs are more consistent with 4 h values (1 h typically 10-20% < 4 h). Therefore pre-cleanup sediment contamination levels defined by the 1998/99 (Brown Bay Grid) data set are likely more reliable than those derived from the earlier 0.5 h data.
- Extraction over 4 h provides the most reproducible data for both CRMs and presumably also control and contaminated sediment samples. Consequently, variation in concentration data measured for sediment over different seasons using 4 h extraction (i.e. in the period 2006-2015) can be considered with high confidence to indicate real differences in the sediment (e.g. changes in sample or site heterogeneity for controls and in contaminant content or availability for impacted sediments) and not as analytical artefacts.

Figure S2. Deviation of ratio PACS-2 : MESS-2/3 (model concentration ratio for sediment samples) from the 4h reference value in the 1 M HCl partial extraction analysis sets. Data for Ag, Sb or Sn are not shown (no reference data for Ag; many of the MESS-2/3 data for Sb and Sn are ≤RL (including 4 h reference values) resulting in all deviations of the ratio in the range -70 to -100%).

#### The effect of extraction time on the 1 M HCl extraction of Casey sediments

Revisiting the work of Snape et al. (2004) allows us to compare data for Casey sediments with the estimates of bias from variable extraction time obtained for the CRMs. A comparison of metal data obtained by 0.5 h and 4 h extractions for a set of sediment samples collected in 1998 is shown in Fig. S3 and the results of a 2 factor PERMANOVA analysis (location and extraction time) presented in Table S2. For most elements there was no discernible effect but for Cr, Fe, Mn and Ni there was a significant difference between the 0.5 h and the 4 h extractions (Table S2, Fig. S3). For Cr, Fe and Ni the effect, in most cases, was a relatively minor increase in the 4 h extraction, but for Mn there was a much larger increase for all samples. There was also a significant interaction term for Cr, Mn and Ni: at some locations there was no difference between 0.5 h and 4 h data but there was at others; and also there were differences among locations for the 4 h but not the 0.5 h extraction. This was consistent with the complex pattern of effects observed for the two CRMs. Similarly, the increase in Cr, Mn and Ni concentration determined by 4 h compared to 0.5 h extraction was by a factor of 2-3 (i.e. a bias in the 0.5 h relative to 4 h data of ‑50 to ‑66%) or even 4x (‑75% bias) in extreme cases.

#### Table S2. Effects of 30 minute versus 4 hour extraction time on 1 M HCl metal extractable concentration in sediments.

|  | Location | Extraction time | Location x Extraction time |
| --- | --- | --- | --- |
| Ag | 0.0001 | 0.2 | 0.3 |
| As | 0.0001 | 0.3 | 1 |
| Cd | 0.0001 | 0.3 | 0.8 |
| Cr | 0.0001 | **0.0001** | **0.05** |
| Cu | 0.0001 | 0.7 | 0.9 |
| Fe | 0.0001 | **0.02** | 1 |
| Mn | 0.0001 | **0.0001** | **0.0001** |
| Ni | 0.0001 | **0.0001** | **0.03** |
| Pb | 0.0001 | 0.9 | 1 |
| Sb | 0.0001 | 0.06 | 0.1 |
| Sn | 0.0001 | 0.9 | 1 |
| Zn | 0.0001 | 0.7 | 0.9 |

Results of two factor PERMANOVA showing P values (from permutation test) for the factors *Location*, *Extraction time* and the *interaction term*.


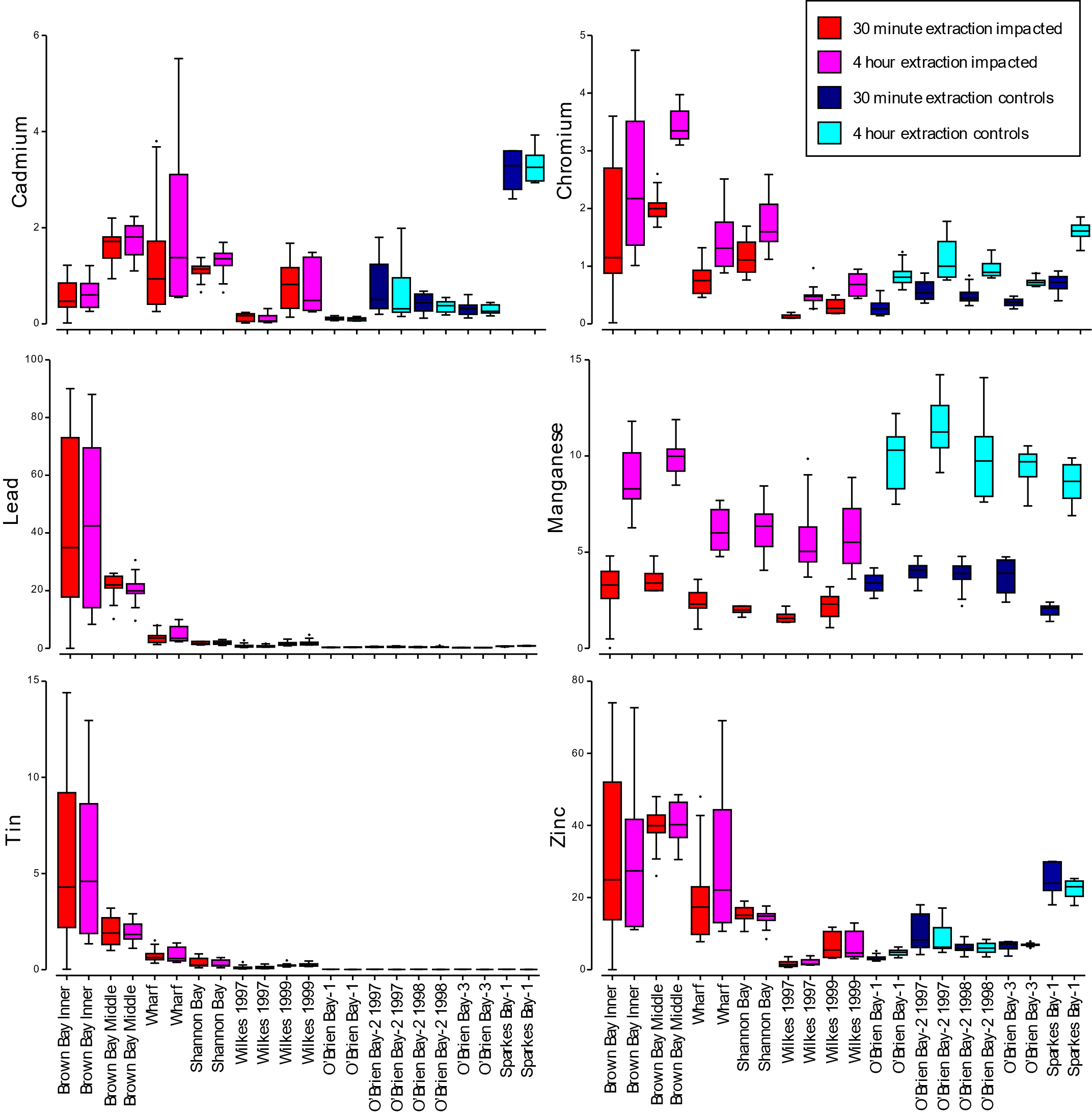


Figure S3: Comparison of 30 minute versus 4 hour 1 M HCl extraction time on metal concentrations (all mg kg^-1^ dry weight). Samples collected in summer 1997/98 survey, with additional samples from summer 1998/99 at Wilkes and O’Brien Bay 2. N = 8.


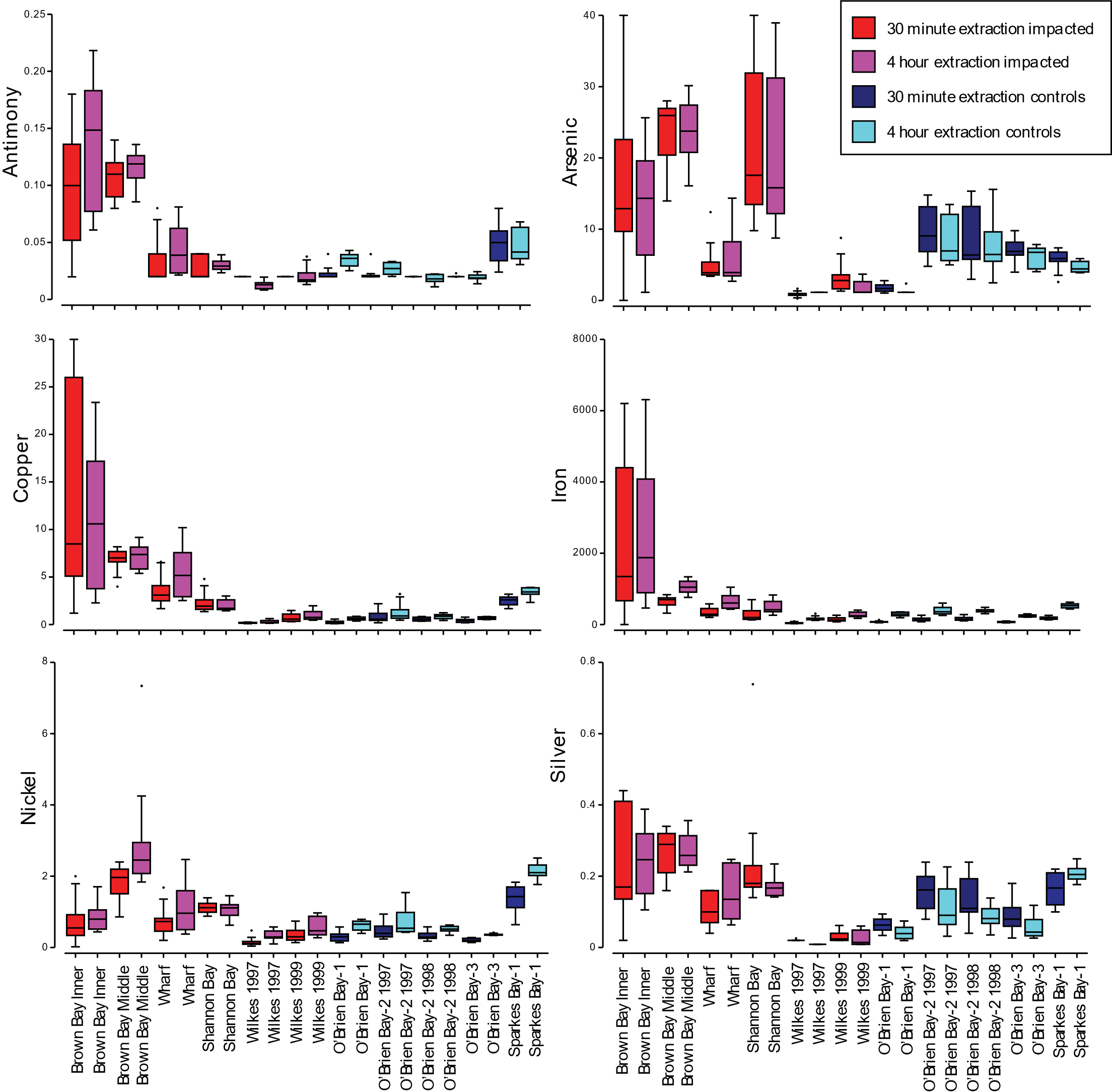


Figure S3 (Continued)

# References

American Public Health Association (APHA). 2005 Standard Methods for the Examination of Water and Wastewater, 21st ed., APHA, AWWA, WPCF, Washington.

Canadian Council of Ministers of Environment (CCME). 2001. Reference Method for the Canada-Wide Standard for Petroleum Hydrocarbons in Soil – Tier 1 Method. ISBN 1-896997-01-5. Publication No. 1310.

Clark, M.W., Davies-McConchie, F., McConchie, D., Birch, G.F. 2000. Selective chemical extraction and grainsize normalisation for environmental assessment of anoxic sediments: validation of an integrated procedure. Science of the Total Environment, **258**, 149-170.

Fryirs, K.A., Hafsteinsdóttir, E.G., Stark, S.C., Gore, D.B. 2015. Metal and petroleum hydrocarbon contamination at Wilkes Station, East Antarctica. Antarctic Science, **27**, 118-133.

Heiri, O., Lotter, A.F., Lemcke, G. 2001. Loss on ignition as a method for estimating organic and carbonate content in sediments: reproducibility and comparability of results. Journal of Paleolimnology **25**, 101-110.

Kersten, M., Förstner, U., 1989. Trace element speciation in biological systems. In: Batley, G.E. (Ed.), Trace Element Speciation: Analytical Methods and Problems. CRC Press, Inc., Florida, 350 pp.

Lambkin, D.C. and Alloway, B.J. 2000. The problem of arsenic interference in the analysis of soils for cadmium by inductively coupled plasma-optical emission spectrometry. Science of the Total Environment, **256**, 77-81.

Malo, B.A. 1977. Partial extraction of metals from aquatic sediments. Environmental Science and Technology,**11**, 277-282.

McBride, M.B. 2011. A comparison of reliability of soil Cd determination by standard spectrometric methods J*ournal of Environmental Qual*ity, **40**, 1863–1869.

Simpson, S. and Batley, G.(Eds.) 2016. Sediment quality assessment : a practical guide. 2nd ed., CSIRO Publishing, 2nd ed., Clayton South, 346 pp.

Snape, I., Scouller, R.C., Stark, S.C., Stark, J.S., Riddle, M.J., Gore, D.B., 2004. Characterisation of the dilute HCl extraction method for the identification of metal contamination in Antarctic marine sediments. Chemosphere 57, 491-504.

Stark, J.S., Snape, I., Riddle, M.J., Stark, S.C. 2005. Constraints on spatial variability in soft-sediment communities affected by contamination from an Antarctic waste disposal site. Marine Pollution Bulletin, **50**, 276-290.

Sutherland, R.A. 2002. Comparison between non-residual Al, Co, Cu, Fe, Mn, Ni, Pb and Zn released by a three-step sequential extraction procedure and a dilute hydrochloric acid leach for soil and road deposited sediment. Applied Geochemistry, **17**, 353-363.

Townsend, A.T. 2000. The accurate determination of the first row transition metals in water, urine, plant, tissue and rock samples by sector field ICP-MS. Journal of Analytical Atomic Spectrometry **15**, 307–314.

Townsend, A.T., Palmer, A.S., Stark, S.C., Samson, C., Scouller, R.C., Snape, I. 2007. Trace metal characterisation of marine sediment reference materials MESS-3 and PACS-2 in dilute HCl extracts. Marine Pollution Bulletin, **54**, 226-246.
